# The homotopic connectivity of the functional brain: a meta-analytic approach

**DOI:** 10.1038/s41598-019-40188-3

**Published:** 2019-03-04

**Authors:** Lorenzo Mancuso, Tommaso Costa, Andrea Nani, Jordi Manuello, Donato Liloia, Gabriele Gelmini, Melissa Panero, Sergio Duca, Franco Cauda

**Affiliations:** 1 GCS-fMRI, Koelliker Hospital and Department of Psychology, University of Turin, Turin, Italy; 2 Focus Lab, Department of Psychology, University of Turin, Turin, Italy

## Abstract

Homotopic connectivity (HC) is the connectivity between mirror areas of the brain hemispheres. It can exhibit a marked and functionally relevant spatial variability, and can be perturbed by several pathological conditions. The voxel-mirrored homotopic connectivity (VMHC) is a technique devised to enquire this pattern of brain organization, based on resting state functional connectivity. Since functional connectivity can be revealed also in a meta-analytical fashion using co-activations, here we propose to calculate the meta-analytic homotopic connectivity (MHC) as the meta-analytic counterpart of the VMHC. The comparison between the two techniques reveals their general similarity, but also highlights regional differences associated with how HC varies from task to rest. Two main differences were found from rest to task: (i) regions known to be characterized by global hubness are more similar than regions displaying local hubness; and (ii) medial areas are characterized by a higher degree of homotopic connectivity, while lateral areas appear to decrease their degree of homotopic connectivity during task performance. These findings show that MHC can be an insightful tool to study how the hemispheres functionally interact during task and rest conditions.

## Introduction

The study of the human brain compels us to challenge many seeming inconsistencies and counter-intuitive observations. Although its generally symmetrical shape, structural and functional asymmetries appear to be important features of the brain organization. These anatomical asymmetries can be found both at the macroscopic and at the microscopic level. Well-known macroscopic asymmetries are the Yakovlevian torque as well as the larger size of the left *planum temporale*^1–4^. Other arguable structural differences among hemispheres have been found in Broca’s area, the central sulcus and the cerebral ventricles^4^. Microscopic asymmetries, in turn, have emerged from the analysis of columnar structure of auditory and verbal areas, such as the greater width and separation of individual columns in the left cortex^5^. These microstructural asymmetries are thought to be associated with typical hemispheric functional specializations^6,7^. Generally speaking, functional asymmetries include a major and dominant involvement of the left hemisphere in language processing^8–12^, and a major involvement of the right hemisphere in spatial attention^13,14^, perceptual control^15^, mental rotation^16^ and spatial coordinate processing^17^.

A central issue in order to understand the relationship between hemispheric symmetries and asymmetries is how the features of segregation and integration can characterize the human brain connectome. Segregation refers to the information processing supported by assemblies of neurons with identical functions and leads to the functional specialization of structurally segregated areas^18,19^. Integration refers to the interaction between specialized brain regions, thus enabling long-range synchronization and information flow^20,21^. So, functional lateralization could derive from the interplay of these two processes, which underpin the division of work between hemispheres^22,23^. These features seem to be at the root of the organization of inter-hemispheric interaction, in particular of the functional connectivity (FC) between mirror regions of the hemispheres. FC is defined as the correlation between neurophysiological indices taken in different parts of the brain^24^; it reflects the intrinsic connectivity that constitutes the brain connectome^25–28^. A relevant part of this neural organization is based on homotopic connectivity (HC), which refers to the functional and structural connectivity between mirror inter-hemispheric areas and has been repeatedly observed at rest^29–34^.

FC appears to be intimately related to structural HC^35–40^, the greater part of which is mediated by many fibers of the corpus callosum (CC)^41–46^. In fact, interhemispheric FC and EEG coherence have been found to be reduced both after callosotomy^47–49^ and in the agenesis of the CC^50–53^. What is more, cytological differences within the CC (regarding the size as well as the myelination of the fibers) correspond anatomically to specific partitions of the cerebral cortex between primary and associative areas^54–56^. Functional and structural distinctions between primary and associative regions are also mirrored by inter-hemispheric coordination. Primary cortices, associated to fast CC fibers, display a strong HC, and associative cortices, connected by slower fibers and often characterized by functional lateralization, show a lower degree of HC^33^. Therefore, HC appears to have different patterns across brain areas, thus leading to a spatial inhomogeneity due to functional purposes.

Following the line of research inaugurated by Stark and colleagues^33^, who studied the correlations of the blood oxygen level dependent (BOLD) signal between large symmetrical brain regions, Zuo *et al*.^57^ calculated the Pearson’s correlations on inter-hemispheric BOLD signal in a voxel-wise fashion, thus obtaining a map of homotopic FC. This technique, named “voxel-mirrored homotopic connectivity” (VMHC), has been efficiently used to assess changes in inter-hemispheric resting state FC (rsFC) in several pathological and abnormal conditions, such as schizophrenia^58–65^, depression^66–71^, addiction^72–75^, bulimia^76,77^, epilepsy^78,79^, insomnia^80^, stroke^81^, early blindness^82^, obesity^83^, acclimatization to high-altitude hypoxia^84^, and passive hyperthermia^85^. The results of these studies provide evidence that HC varies between brain areas and is influenced by many alterations of brain physiology, including ageing^57^. Indeed, age-related changes have been found in VMHC, with sensorimotor regions showing increases and associative regions revealing decreases in HC of aging individuals^57^. Another demographic variable displaying effects on VMHC is sex, with females and males exhibiting regional differences in HC values^57,86^.

A whole-brain meta-analytic study of the human connectome was carried out by Toro and colleagues^87^, using task-based co-activations as the meta-analytic counterpart of rsFC, thus confirming the existence of strong HC in the brain. Furthermore, a seed-based meta-analytic technique inspired by the same principle, known as the “meta-analytic connectivity model” (MACM)^88^, demonstrates that connectivity can be found in task-based co-activations between brain areas, revealing the same networks found in rsFC^89–94^. Therefore, MACM can be seen as the meta-analytic correspondence of the seed-voxel correlation^25^. However, though VMHC is a valid technique for functional data analysis, we still lack a meta-analytic approach in order to investigate HC on functional datasets.

The present study aims to address this issue by developing a meta-analytic approach for HC, and applying it to the functional dataset of BrainMap, which is an online database that currently includes neuroimaging experimental results from more than 3000 studies, organized in a multidimensional keywords system of meta-data^95–97^. Although meta-analytical studies, such as MACM or activation likelihood estimation (ALE)^97–100^, extensively use this keyword system in order to focus on a particular research domain, we took into consideration the entire normal-mapping activation database, as Toro and colleagues did^87^. Of note, Smith and associates^101^ carried out an independent component analysis on the same entire database, obtaining a set of components similar to the canonical resting state networks, thus confirming the correspondence between task-based co-activations and connectivity^102^.

These similarities allow us to propose a method in which brain HC is emulated by homotopic co-activation. We carried out the analysis on the entire functional BrainMap database of healthy subjects in order to acquire the largest possible set of data, with the intention to validate our new “meta-analytic homotopic connectivity” (MHC) technique. Finally, the obtained results have been confronted with those of a VMHC computed on a large dataset, part of the 1000 Functional Connectomes Project (FCP)^103,104^. This comparison aimed at demonstrating not only the similarity between the two techniques but also at investigating how HC changes between rest and task.

## Materials and Methods

### Selection of studies

For our meta-analysis we compiled a pool of neuroimaging experiments from the BrainMap database^95,97^. As illustrated in the User Manual, BrainMap is an open access database of published human neuroimaging experiments that uses a hierarchical coding scheme to report coordinate-based results (x, y, z) in stereotaxic brain space^96^. In order to identify eligible experiments, we queried the entire functional dataset of BrainMap (January 2018). Specifically, using the software package ‘Sleuth 2.4’ (http://www.brainmap.org/sleuth/) we constructed the search algorithm as follows:

*[Experiments Context IS Normal Mapping] AND [Experiments Activation IS Activations Only] AND [Subjects Diagnosis IS Normals]*.

The data downloaded using these search keys were subsequently individually checked in order to ensure that the all the following inclusion criteria were fulfilled: (i) they were original studies published in a peer-reviewed English language journal; (ii) they used any activation paradigm for functional imaging; (iii) they adopted a whole-brain analysis (i.e., field of view not confined to a restricted region of interest); (iv) they included only normal subjects; (v) foci of activation were reported in Talairach/Tournoux (TAL) or in Montreal Neurological Institute (MNI) stereotaxic space.

On the grounds of this criteria, we included in the meta-analysis 2370 articles, for a total of 13148 experiments, 112 paradigm classes and 68152 subjects (see also Supplementary Table S1). Therefore, coordinate-based results were extracted from each selected experiment and using the Lancaster’s icbm2tal transformation^105,106^ we converted results from MNI into TAL stereotaxic space.

Concerning the selection phase, we followed the Preferred Reporting Items for Systematic Reviews and Meta-Analyses (PRISMA) Statement international guidelines^107^ (see Supplementary Fig. S1 for the overview of the selection strategy).

### Activation likelihood estimation

The activation likelihood estimation (ALE)^98,99,108^ was performed to produce the data that were inserted in the MHC algorithm. In the ALE, a 3-dimensional Gaussian probability distribution is constructed around every focus of each experiment, as follows:

$$p(d)={\frac{1}{{\sigma }^{3}\sqrt{{(2\pi )}^{3}}}}^{\frac{d}{2{\sigma }^{2}}}$$

In the formula *d* is the Euclidean distance between the voxel and the given focus, *e* is the spatial uncertainty; the standard deviation is determined through the full-width half-maximum (FWHM) as:

$$\sigma =\frac{FWMH}{\sqrt{8\,\mathrm{ln}\,2}}$$

We produced a modeled activation (MA) map for every experiment as the union of the Gaussian probability distributions derived from the foci of every experiment. Finally, a final ALE map was obtained from the union of all the MA maps. As recommended by Eickhoff *et al*.^98,109^, statistical significance was determined by cluster-level inference. A null distribution of cluster sizes was determined simulating a long series of experiments and calculating a corresponding ALE map. A threshold P value was derived from the resulting score histogram.

### Offset adjustment

If a focus taken from an experiment is placed near to the brain midline, the MA produced around it is likely to cover some portions of both hemispheres, extending from the one containing the focus to the other one. This could generate spurious results, for instance raising the probability values in a region that is proximal to the foci but probable not co-activated. To deal with this potential problem we adjusted the offset value near the median line. On the basis of the mean spatial uncertainty of this kind of meta-analytic data, we predicted that, on average, the Gaussian cloud may extend around 12 millimeters around the focus. Therefore, we decided to compensate an area of 24 mm of width placed along the midline through a weight decreasing function proportional to $\frac{1}{{d}_{ij}}\,$, in which *i* is the midline and *j* is the given voxel. This would attribute to the voxel a progressively minor interference as they are placed further from the midline.

### MHC calculation

To identify whether or not the probability of the activation of a given area is statistically associated with that of its homologue in the contralateral hemisphere, we devised a novel method able to construct a map of HC starting from meta-analytic data.

The first step consisted in partitioning the ideal brain derived from our ALE data by means of an anatomical atlas produced with the symmetrization of the Talairach Daemon^110^ (TD) with a resolution of 2 mm^3^. The second step was to produce an NxM matrix in which each row represents an experiment and each column represents an anatomical area. For each experiment we reported an area as being activated if the MA map of the experiment induced 20% or more voxels in that area to be statistically significant. Then, we calculated the co-activation strength between the homotopic regions through the Patel’s κ index^111^. A probability distribution of each couple co-activation can be constructed from a Bernoulli generation model of data. Given two areas *a* and *b*, it is possible to define their co-activation by the means of the probability of the following cases: (i) *a* and *b* are both activated (ii) *a* is activated and *b* is not (iii) *a* is not activated and *b* is activated (iv) *a* and *b* are both not activated:

$${\theta }_{1}=P(a=1,\,b=1)$$

$${\theta }_{2}=P(a=1,\,b=0)$$

$${\theta }_{3}=P(a=0,\,b=1)$$

$${\theta }_{4}=P(a=1,\,b=0)$$

From Table 1 is possible to derive the marginal probabilities.

**Table 1:** Marginal probabilities between activated and non-activated regions.

**Region** ***b***	**Region** ***a***			
		Activated	Non-activated	
	Activated	θ_1_	θ_3_	θ_1_ + θ_3_
	Non-activated	θ_2_	θ_4_	θ_2_ + θ_4_
		θ_1_ + θ_2_	θ_3_ + θ_4_	1

The Patel’s κ calculates the connectivity as follows:

$$\kappa =\frac{({\theta }_{1}-E)}{D({\rm{\max }}({\theta }_{1})-E)+(1-D)(E-\,{\rm{\min }}\,({\theta }_{1})}$$

where

$$\begin{array}{rcl}E & = & ({\theta }_{1}+{\theta }_{2})({\theta }_{1}+{\theta }_{3})\\ {\rm{\min }}\,({\theta }_{1}) & = & {\rm{\max }}(0.2{\theta }_{1}+{\theta }_{2}+{\theta }_{3}-1)\\ {\rm{\max }}\,({\theta }_{1}) & = & {\rm{\min }}({\theta }_{1}+{\theta }_{2},{\theta }_{1}+{\theta }_{1}+{\theta }_{3})\end{array}$$

The numerator of the fraction is the difference between the probability that *a* and *b* are co-activated and the probability that their activation is statistically independent. The denominator is a weighted constant of normalization. *Min*(*θ*1) represents the maximum value of conjoint probability *p*(*a*,*b*) given *p*(*a*) and *p*(*b*), and *max*(*θ*1) represents the minimum value of *p*(*a*,*b*) given *p*(*a*) and *p*(*b*). Patel’s κ index values range from −1 and +1. Values close to |1| denote high connectivity. Patel’s κ statistical significance can be assessed through a simulation based on a Monte Carlo algorithm. The Monte Carlo method estimate *p*(*κ*|*z*) by sampling a Dirichlet distribution and determining the proportion of the samples in which *κ* > *e*, where *e* is a threshold of statistical significance.

### Atlas and threshold evaluation

A confounding aspect of our MHC method arises from the arbitrary selection of the percentage of significantly active voxels to induce a region to be considered as activated. Therefore, we repeated the MHC calculation with the method explained in the previous section, but varying the threshold to 0%, in which a single active voxel is able to drive the entire region to be active, and to 40%, in which the activation criteria is much more conservative. The two maps produced with these thresholds were then correlated with the MHC calculated with the 20% threshold.

Another possible limitation comes from the choice of the atlas, and, in particular, from the seize of its volumes. As an area is considered activated if at least 20% of its voxels are included in a MA, the size of a couple of bilateral regions could influence their Patel’s κ. In particular, smaller areas are more likely to appear active in that a single MA could cover most of their voxels, while bigger regions could need more than one MA to cover the 20% of their voxels. On the other hand, a bigger area is more likely to include one or more MAs. The possibility that bigger volumes could be biased to be more activated do not seem a real concern, because the threshold of activation of a region is calculated as the percentage of its significantly active voxels, thus the size of each area would have already been weighed. However, bigger areas could be characterized by a higher statistical power than smaller ones due to their higher number of voxels to be modelled as active. This latter problem could be corrected using a Voronoi tessellation that divides the brain in regions having each the same number of foci, as implemented in previous meta-analyses^90,92^. However, this solution has the negative consequence to create areas of very different volumes with no relationship with anatomic and functional boundaries. Moreover, as the foci are not symmetrically distributed, this tessellation would produce so an asymmetrical template, making impossible to match two regions considered to be as homotopic. Therefore, a different approach has been adopted.

We created two control parcellations characterized by regions having the same number of voxels, using the method proposed by Fornito and colleagues^112^. Briefly, every region of our original symmetrical atlas was randomly partitioned in a series of volumes having the same number of *k* voxels. Homogeneous atlas 1 was partitioned in 128 volumes having *k* = 997 voxels, and homogeneous atlas 2 was partitioned in 512 volumes having *k* = 250 voxels. Parcellations with higher values of *k* would produce too big regions, losing spatial resolution of the results. Smaller values of *k* would produce a result more similar to a voxel-wise technique, losing the advantage of the higher statistical power of using an atlas. These two atlases do not suffer of regional biases introduced by different size regions, and could also be used to control the effect of the size of the volumes, as they represent a condition of reasonably big and reasonably small partitioning, respectively. Therefore, the atlases produced with the two selected *k* were used to calculate the MHC with the method previously described, and their results correlated with the MHC map obtained with the TD atlas.

### Resting state data

The resting state data used to calculate the VMHC are part of the 1000 FCP^103^. Specifically, we chose the Beijing dataset, which contains 198 subjects (122F, 76M) of age between 18 and 26 years. The scan was performed with a repetition time (TR) of 2 seconds, 225 time points were acquired in 7.5 minutes of scanning, and 33 slices were acquired for each volume, with a spatial resolution of 3.125 × 3.125 × 3.6 millimeters. For each subject, a T1-weighted anatomical scan was also acquired, with a spatial resolution of 1.33 × 1 × 1 millimeters. Given the sex effect on VMHC values^57,86^, we chose to exclude 46 randomly chosen female subjects to equilibrate the two sex groups to a 1:1 sex ratio, hypothetically comparable to that of the global population.

### VMHC calculation

The VMHC and the associated preprocessing of anatomical images was carried out with DPARSFA^113^ (http://rfmri.org/DPARSF) which is based on SPM12 (http://www.fil.ion.ucl.ac.uk/spm) and the toolbox DPABI^114^ (http://rfmri.org/DPABI) on the Beijing dataset of the FCP^103^. The preprocessing pipeline was composed as follows: (i) although the first 5 timepoints were already removed by the submitter of the dataset, 10 other volumes were discarded; (ii) a slice timing correction was applied; (iii) the functional images were realigned to correct head movement; (iv) each anatomical T1 image was co-registered to its functional image; (v) the anatomical images were segmented using DARTEL^115^. The affine regularization in segmentation was set to be optimized for East Asian brain; (vi) the Friston 24 model^116^ was used to out-regress movement artifacts, and white matter and cortico-spinal fluid were estimated with an *a priori* mask and used as nuisance regressors; (vii) functional images were co-registered to a mean anatomical template generated in the segmentation step.

The VMHC was performed and set for the normalization to symmetric template. No smoothing passage was performed before or after the VMHC calculation. Five subjects (3F, 2M) were discarded after a quality assessment step. The remaining 147 z-points-transformed individuals’ VMHC maps (73F, 74M) were then averaged with flsmaths utility of FSL (https://fsl.fmrib.ox.ac.uk/fsl/fslwiki/FSL)^117,118^ so as to obtain the VMHC group map. Finally, the VMHC map was co-registered to the MHC map in a Talairach space with a resolution of 2 mm^3^, so as to allow the calculation of their Pearson’s correlation coefficient.

### Leave-one-out correlation analysis and map of the differences

To analyze the regional differences between the two methods, we devised an analysis “leave-one-voxel-out” to assess the voxels’ contribution to correlation (VCC) between the two maps. Each pair of corresponding voxels ${v}_{{i}_{1}}$ and ${v}_{{i}_{2}}$ was removed from both maps, and after every removal the *r* of Pearson between the two maps was re-computed. The difference ${r}_{GLOBAL}-{r}_{{v}_{i}REMOVED}$ between the original value of *r* and the value obtained removing the couple of voxels ${v}_{{i}_{1}}\,$and ${v}_{{i}_{2}}\,$was assigned to voxel *v*_*i*_. The procedure was repeated for *v*_*j*_, *v*_*k*_ …, then the difference values were normalized, obtaining a voxel-wise evaluation of local contribution to the global correlation. Negative VCC values indicate voxels whose removal determines an increase of the correlation, meaning that they do not contribute positively to the global *r*, or that they contribute to a hypothetical negative correlation. Conversely, positive VCC values correspond to voxels contributing to a positive correlation between the two maps, whose removal produces a *r*_*GLOBAL*_ decrease.

The VCC can inform us about what regions contribute to the similarity and the dissimilarity of the two maps, but it cannot inform whether their dissimilarities derive from stronger values of the MHC or the VMHC map. Since we were interested on what regions appear to be more homotopically connected in our meta-analytic technique compared to the VMHC and vice-versa, the values of MHC and VMHC were both transformed in z-points to make the two measures (the Patel’s κ and the Pearson’s *r*) to be more similarly distributed and therefore legitimately comparable despite the diversity of their nature. Then, the two matrices were subtracted one to the other (*MHC*–*VMHC*) to obtain a statistical map which represents the regional dominance of one technique to the other. Positive values characterize voxels in which the MHC is stronger than the VMHC, while negative values characterize voxels in which the VMHC is stronger than the MHC. This map is a descriptive parameterization of the difference between the results we obtained with the meta-analytic and the resting state techniques, and should be considered as a mere portrayal of the data produced by the two techniques, as no inferential statistics are computed. The operation of subtraction should be considered as the mathematical implementation of a mere comparison between the two maps. Although this sort of comparison may be considered debatable, in this case its validity is in part supported by the similarity of its results to those of the VCC. As it will be shown in the Results section, the areas of negative values of the VCC map, which could be considered as areas of difference between the techniques, are substantially overlapping with regions with extreme values in the difference map, which are characterized by stronger values in the MHC or in VMHC map.

### Behavioral analysis

To understand the cognitive function of a superior temporal area that showed unexpected positive values of difference between MHC and VMHC, a post-hoc behavioral analysis^119^ was performed. The assumption of this technique is that the spatial distribution of activation foci related to a particular subdomain of the BrainMap database reflects the true probability distribution function of that cognitive functional activations, therefore it associates a spatial coordinate to the probability of being activated by each of the BrainMap behavioral subdomain^119^. The z-points-normalized statistical map of differences was thresholded with the *Threshold to ROI* function of Mango (http://ric.uthscsa.edu/mango/) so as to create a region of interest including only the voxels with values higher than 1. A set of regions of interest (ROIs) was manually placed to include only the voxels of a bilateral superior temporal stripe that survived the threshold. The threshold-ROIs and the manual-ROIs where then intersected to select the voxels of interest. This final ROI was fed to the behavioral analysis plug-in in Mango, which searched in the BrainMap database for the behavioral domains and subdomains statistically associated with the selected region.

## Results

### Comparisons of thresholds and atlases

The use of different thresholds to indicate a region as active produced very similar results (see Fig. S2 in the Supplementary Materials). The correlation between the map produced with the intermediate 20% threshold and the two extreme thresholds of 0% and 40% were *r* = 0.78 and *r* = 0.77, respectively. Considered the consistency of these results, the intermediate threshold was chosen to be used in the subsequent analyses.

**Figure 1:**
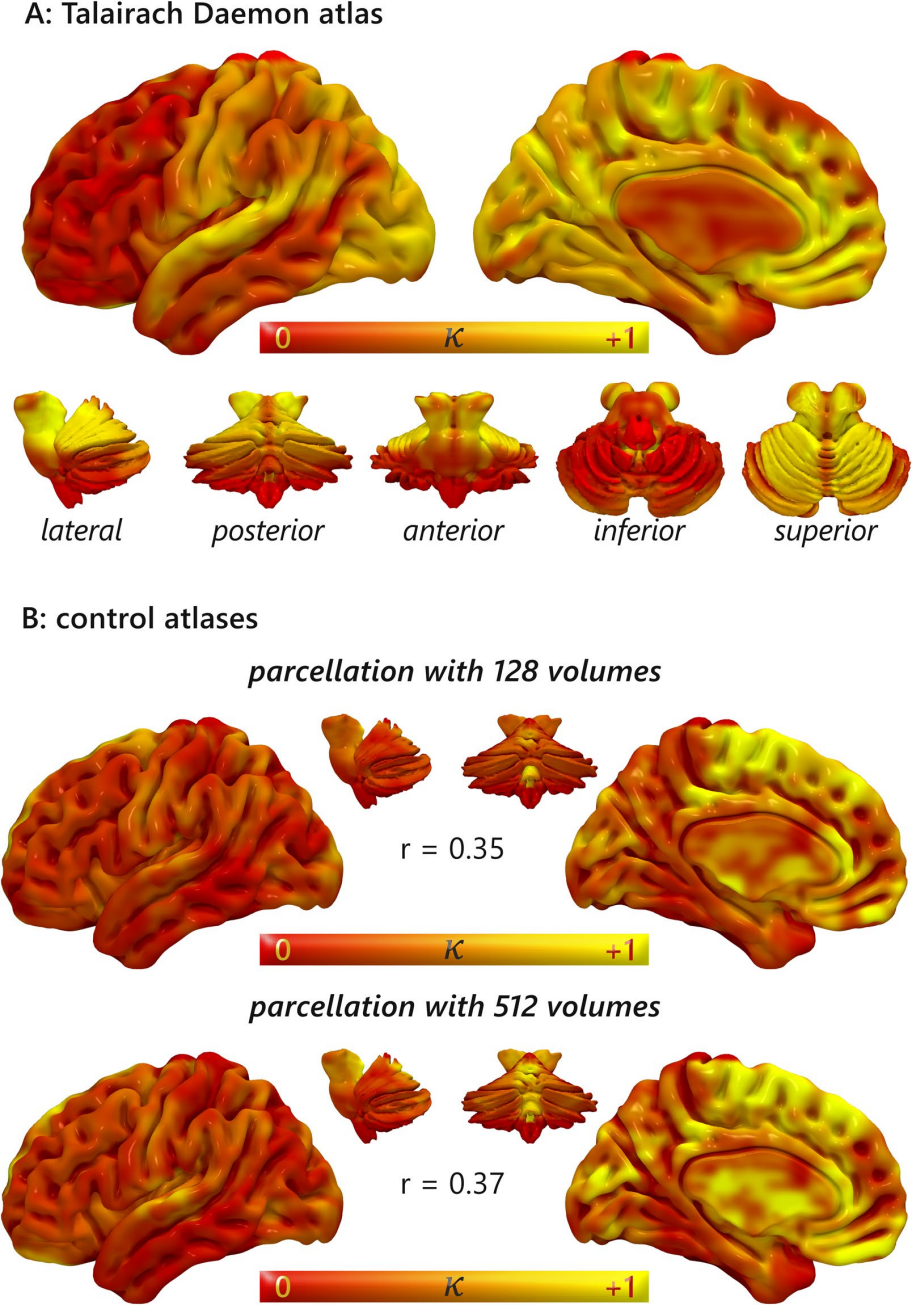
Surface mapping of meta-analytic homotopic connectivity Colors from red to yellow indicate the strength of homotopic connectivity. Despite the Patel’s κ can range from −1 to 1, the MHC shows no negative values, therefore they are not displayed in the color scale. (**A**) Results obtained using the Talairach Daemon atlas. (**B**) Results obtained using the two uniformly parcellated atlas with k = 997 (up) and k = 250 (bottom).

**Figure 2:**
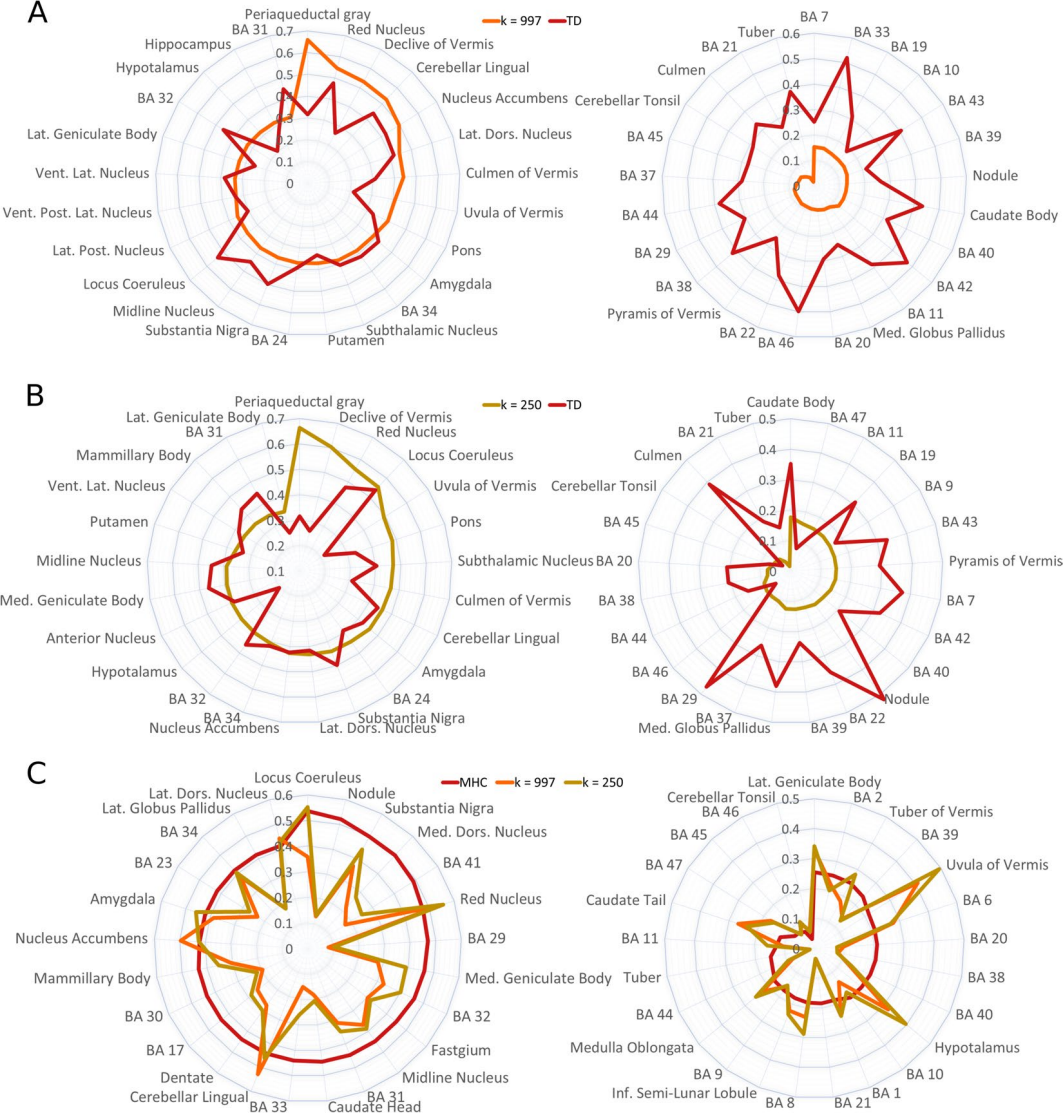
Comparison between the results with the three atlases used. (**A**) The 25 regions more connected and less connected using the homogeneous atlas of 128 volumes compared to the Talairach Daemon atlas. (**B**) The 25 regions more connected and less connected using the homogeneous atlas of 512 volumes compared to the Talairach Daemon atlas. (**C**) The 25 regions more connected and less connected using the Talairach Daemon atlas compared to the other two atlases.

The different atlases produced similar results but with notable differences. The correlation between the result obtained with the TD atlas and the 128 volumes (*k* = 997) atlas was *r* = 0.35, and that between TD and the 512 volumes (*k* = 250) atlas was *r* = 0.37 (Fig. 1). Mean κ values for 89 bilateral regions can be found in Supplementary Table S2. Between the most connected regions obtained with both the homogeneous atlases there are the medial cortices, such as Brodmann areas (BAs) 24, 31, 32, 34, and several cerebellar and subcortical nuclei such as the culmen of vermis, the subthalamic nucleus and the putamen. Between the less connected regions both maps show BA 19 of the occipital lobe, BAs 20, 21, 22, 37, 38 and 42 of the temporal lobe, BAs 7, 29, 39 and 40 of the parietal lobe, BAs 11, 43, 44, 45, 46 of the frontal lobe. Although in many regions the two approaches lead to convergent results, such as BAs 34, 21 44, 45 and 46, the two homogeneous parcellation produced results that are more similar between them than with the anatomical atlas, (Fig. 2). This observation suggest that the size of the volumes do not introduce a significant bias in the results, but the choice between an anatomical atlas or an automated parcellation does.

### The meta-analytic homotopic connectivity computed on an anatomical atlas

With regard to the MHC map obtained with the TD atlas, the most co-activated areas were the following: BAs 32 and 33, which are part of the anterior cingulate cortex (ACC); BA 31, which is part of the PCC, along with BAs 29 and 30, which form the retrosplenial cortex (Rsp); BA 41 in the temporal lobe; and several subcortical nuclei of the basal ganglia, thalamus and cerebellum (Fig. 2C, for more details see Supplementary Table S3). As can be observed in Fig. 1A, medial areas show higher co-activations compared to lateral areas, and occipital and superior temporal regions are the most co-activated between the lateral areas. Areas characterized by minor co-activations were the following: BAs 46, 47, 44 and 45 in the lateral prefrontal cortex; BAs 8, 9, 10 and 11 in the lateral and medial prefrontal cortex; BA 6 in the frontal lobe; BAs 38, 20 and 21 in the temporal lobe, along with the hypothalamus. In general, frontal lobe and other associative cortices appear less homotopically co-activated than primary cortices (Fig. 2C, for more details see Supplementary Table S3).

### Correlation between MHC and VMHC

To evaluate how much our results can be compared with those obtainable with a VMHC performed on functional data, and therefore how much homotopic co-activations can reflect resting state HC, we calculated the VMHC on the Beijing dataset of the FCP^103^ (Figure S3 in the Supplementary Materials) and we determined the correlation between the matrices containing the outputs of the two techniques; all the non-brain voxels were excluded from the calculation. The two maps had a correlation of *r* = 0.51. The correlation between the VMHC and the two MHC maps produced with the homogeneous parcellations are *r* = 0.31 for the 128 volumes atlas and *r* = 32 for the 512 volumes atlas, respectively. Therefore, the method producing the most similar results to the pre-existing technique is the one using an anatomical atlas rather an automatic parcellation. For this reason, the following detailed comparison between the MHC and VMHC maps will be conducted with the anatomical atlas results.

### Leave-one-voxel-out correlation between MHC and VMHC

**Figure 3:**
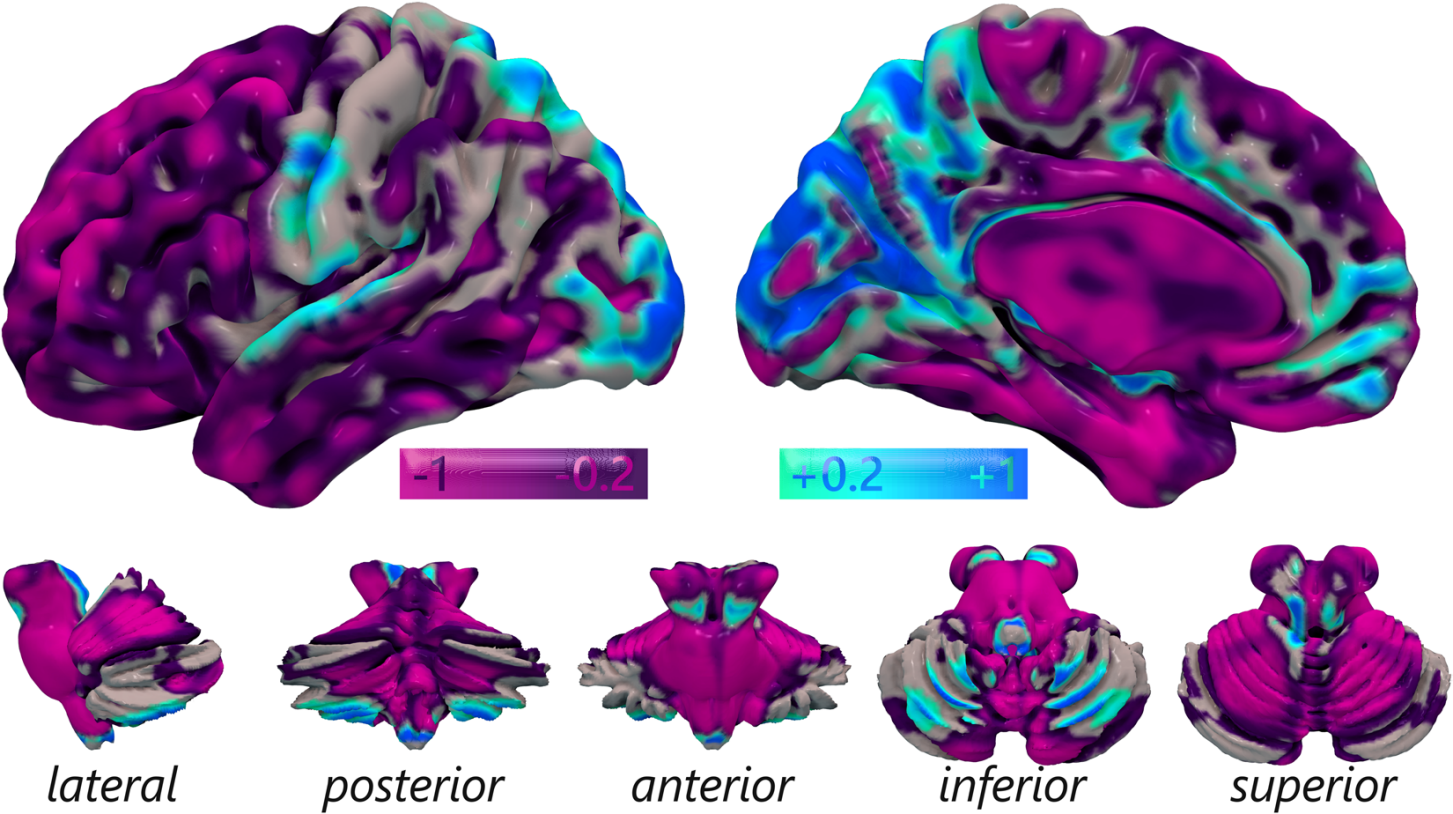
Surface mapping of the leave-one-voxel-out correlation between MHC and VMHC. Colors from dark purple to light purple indicate the voxels less contributing to the global positive correlation, or more contributing to a negative correlation, and could be considered as areas of difference between the MHC and VMHC results. Colors from blue to light blue indicate the voxels more contributing to the global positive correlation, and could be considered as regions of concordance between the two techniques. Non-significant values are not shown.

The VCC analysis produced a map of regions characterized by different levels of contribution to the global correlation. In other words, some regions can be considered as areas of convergence between the two maps (positive values), while others can be seen as areas of divergence (negative values). Areas of convergence are the BAs 17, 18 and 19 in the occipital lobe, the precuneus, BAs 23, 29, 30 and 31 that form the PCC/Rsp, BAs 41 and 42 in the temporal lobe, the nucleus accumbens and several thalamic nuclei. Areas of divergence are the BAs 20, 35, 36 and 38 in the temporal lobe, BAs 8, 9 and 10 of the frontal lobe, and several subcortical and cerebellar nuclei (Fig. 3 and Supplementary Table S3).

### Difference map between MHC and VMHC

**Figure 4:**
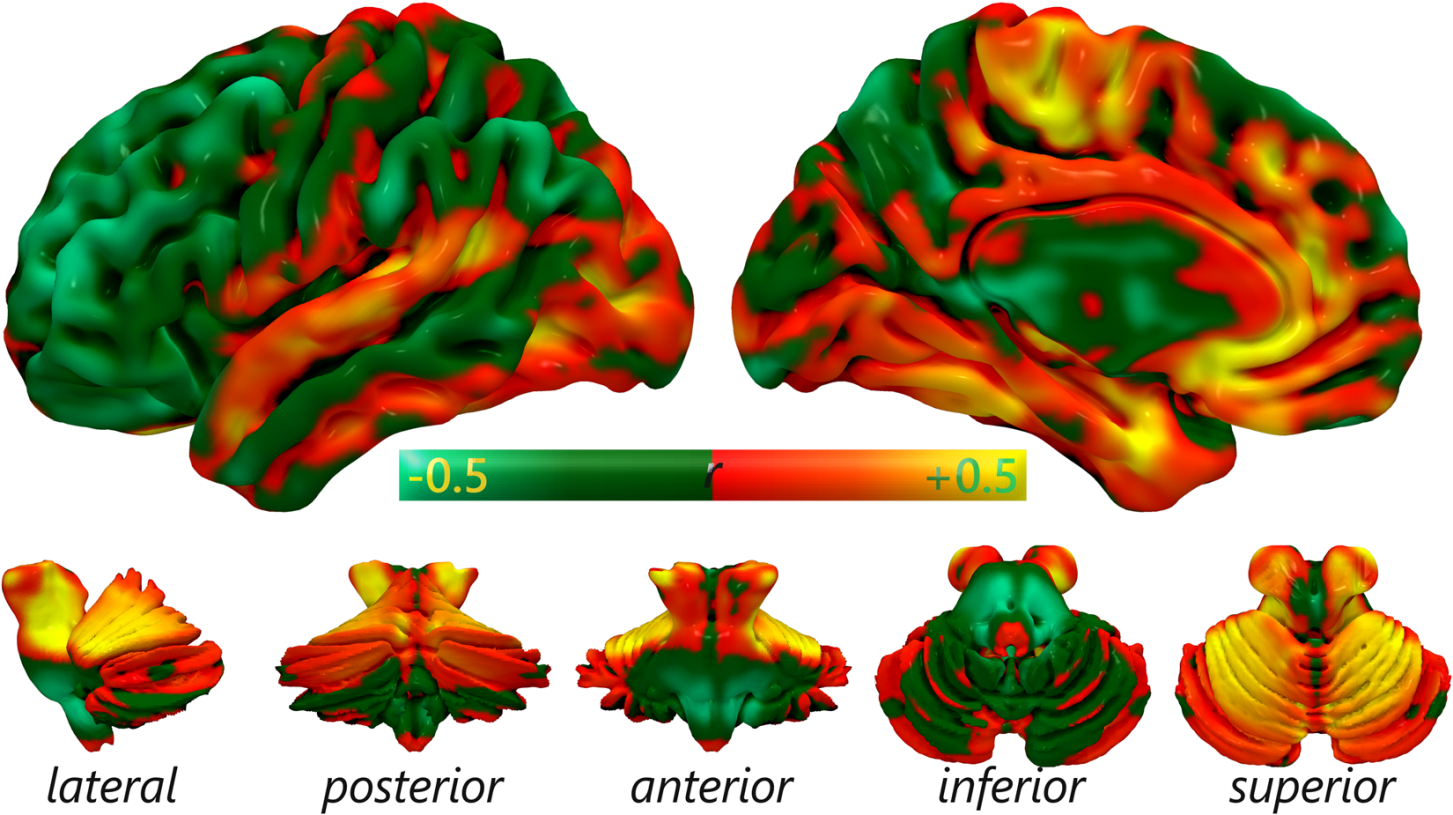
Surface mapping of the difference between MHC and VMHC. Colors from dark green to light blue indicate the relative strength of the VMHC map compared to MHC map. Colors from red to yellow indicate the relative strength of the MHC map compared to VMHC map.

**Figure 5:**
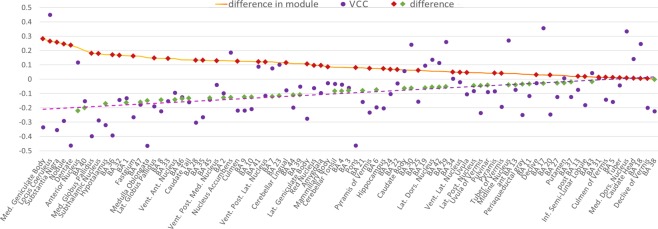
Relationship between the voxels’ contribution to correlation (VCC) map and the difference map, as depicted by the mean values of brain areas. On the y axis, the brain areas are ordered by their decreasing mean absolute values of difference: areas displaying stronger value on the MHC or VMHC maps are placed on the right, areas with similar values in the two techniques are placed on the left. (A decreasing solid orange line represents their mean absolute values of the difference map. Positive and negative values of mean difference are showed as red and green rhombuses, respectively, indicating whether each region is more involved in the MHC or in the VMHC map. Purple dots represent the contribution to correlation of each area as calculated by the VCC analysis. The linear interpolation of these values is depicted as a purple dotted line.

The divergence of a voxel as revealed by a negative value in the VCC analysis can be explained by a strong difference between the MHC and VMHC results. To understand if a region of divergence is characterized by higher meta-analytic values compared to resting state data, or the other way around, we subtracted the two maps. This approach is justified by the similarity between the difference map and the VCC map. Areas of strong difference between MHC and VMHC, colored in yellow and light blue in Fig. 4, are substantially similar to regions of divergence, colored in purple in Fig. 3. The coherence between the VCC and difference maps is also evident in Fig. 5, where the mean values of the VCC map for each brain area are plotted with the mean difference values. The mean difference absolute values correlate negatively with the average VCC values (*r* = −0.33), as illustrated by Fig. 5 as the inverse slopes of the line formed by the absolute values of difference and the line of best fit of the VCC values.

**Figure 6:**
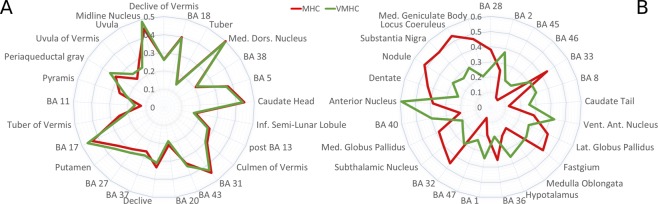
Comparison between regional mean HC as measured by MHC and VMHC. (**A**) The 25 regions showing the highest similarity. (**B**) The 25 regions showing the highest difference.

**Figure 7:**
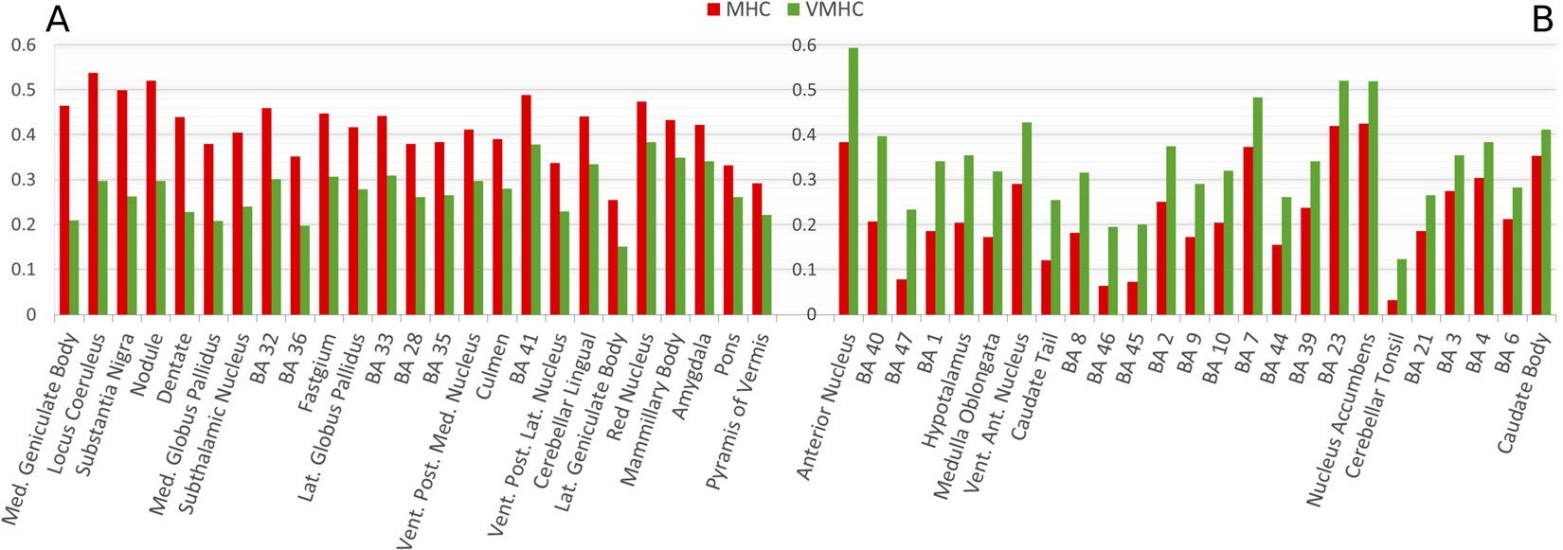
Areas showing more connectivity in the MHC map or in the VMHC map, ordered by decreasing difference between the results. (**A**) The 25 regions characterized by stronger HC in the MHC compared to VMHC. (**B**) The 25 regions characterized by stronger HC in the VMHC compared to MHC.

Some minor regional differences are evident in the difference map (Fig. 4). The most similar areas were the following: temporal regions such as BAs 20, 38, 37 and 27; occipital regions such as BAs 17 and 18; BAs 5 and 31 in the parietal cortex, and posterior insula (more details are illustrated in Fig. 6A). The most differentiated areas were BAs 8, 45, 46 and 47 of the prefrontal cortex; BA 32 and 33 of the ACC, BAs 1, 2 of the primary somatosensory cortex, BA 40, along with several subcortical nuclei (for more details see Fig. 6B). Compared to the VMHC map, the more connected regions in the MHC map are mainly the subcortical nuclei, as well as some cortical areas, such as BAs 32 and 33 of the ACC, BAs 35, 36, 41 and 28 in the temporal lobe (Fig. 7A). Conversely, compared to the MHC map, the more connected regions in the VMHC map are several cortical areas, such as BAs 6, 8, 44, 45, 46 and 47 in the frontal lobe; BAs 1, 2 and 3 in the postcentral gyrus; BAs 23, 7 and 40 in the parietal lobe; BA 21 in the temporal cortex, as well as portions of the subcortical nuclei (Fig. 7B). In general, associative areas in the lateral frontal and middle parietal cortices are characterized by more HC in the VMHC than in the MHC, while areas surrounding the anterior and medial cingulate gyrus, inferior medial temporal lobe (MTL) and a region corresponding to the superior temporal gyrus (STG) are characterized by stronger HC in the MHC than in the VMHC (Fig. 4).

### Behavioral Analysis

**Figure 8:**
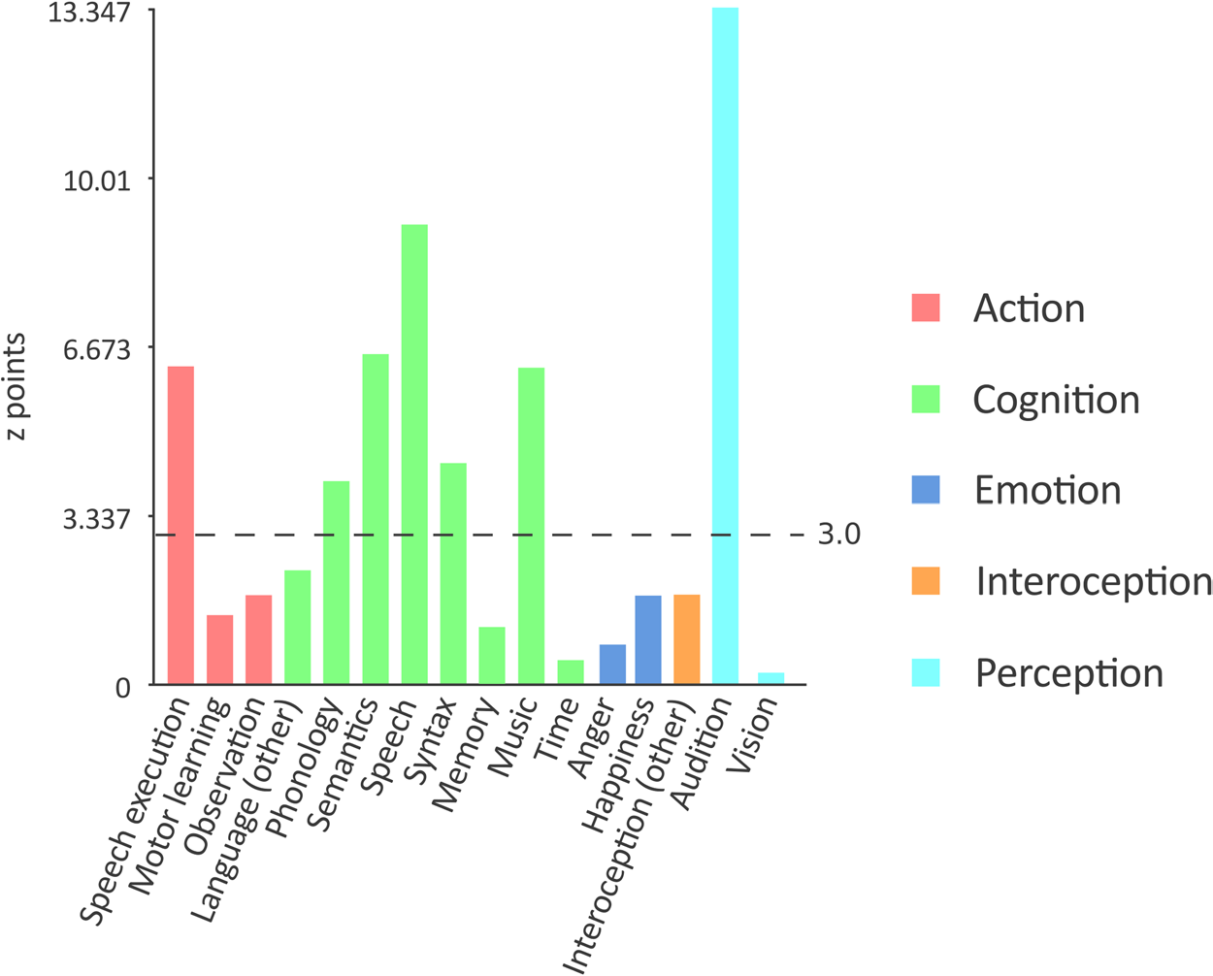
Behavioral analysis of the bilateral superior temporal gyrus (STG). The dashed line indicates the threshold of statistical significance. The same analysis, executed both on the left and on the right STG, produces similar results, with weaker linguistic functions for the right STG (not shown). Significant z-points values: speech execution = 6.268; phonology = 4.041; semantics = 6.543; speech = 9.126; syntax = 4.379, auditory perception = 13.347 and musical functions = 6.259.

As the MHC involvement in the STG is one of the most evident results emerging from the observation of Fig. 3, we investigated its function with a behavioral analysis. As depicted in Fig. 8, the bilateral ROI including the superior temporal voxels characterized by a preference toward the MHC map are related to linguistic (z-scores: speech execution = 6.268; phonology = 4.041; semantics = 6.543; speech = 9.126; syntax = 4.379), auditory (auditory perception = 13.347) and musical (=6.259) functions.

## Discussion

### The meta-analytic homotopic connectivity

The development and application of our technique provides a new tool to calculate the homotopic connectivity starting from meta-analytic data so as to construct a homotopic co-activation map that varies from area to area (Fig. 1), thus indicating different levels of homotopic co-activation. It is worth noting that its outcomes appear relatively stable to the different thresholds used, increasing its validity in investigating HC regardless of the arbitrariness of this methodological choice. The variability in the resulting map echoes well-known patters of FC. For example, many medial areas are characterized by more HC than lateral areas, despite the application of the offset adjustment on MAs that are close to the midline. This is not surprising, since the negative effect of the Euclidean distance on HC has been already observed by Stark and colleagues^33^. Indeed, between the most co-activated areas (independently of the atlas used) there are many subcortical and cerebellar structures, which are placed near the midline, or many medial cortices such as BAs 32 and 34 (Fig. 2). Other strongly co-activated areas with the TD atlas include the BAs 17 and 41, that are the primary visual and auditory cortices. Although the primary motor and somatosensory cortices are not between the most co-activated areas with none of the atlases used, they are more co-activated than heteromodal regions, many of which show a low mean Patel’s κ value (Fig. 2). These findings, too, are consistent with the study by Stark and associates^33^, since they found an effect of stronger HC in primary cortices compared to heteromodal regions.

Indeed, the least connected areas (such as BAs 6, 8, 9, 10, 44, 45, 46 and 47 in the prefrontal cortex) are all associated with memory^120–124^, working memory^122,125–128^, and language^129–138^. Specifically, the lateral and dorsomedial prefrontal cortices are part of the executive-control network, which is essentially associated with working memory and control function^139,140^. The dorsal attention network is dedicated to the top-down control of attention^141^ and encompasses the frontal eye field area, which likely involves both BAs 8 and 6^142^. Also BAs 20, 21 and 38 in the temporal lobe, which also show low Patel’s κ mean values, have been associated with memory^143–146^ and, especially, with language^147–155^. BAs 21 and 38 are considered part of the DMN^156,157^, and have been found to be activated by empathy and perspective-taking tasks^158,159^. These considerations support the idea that the least homotopically co-activated areas are involved in hierarchically high cognitive functions, as pointed out by Stark and colleagues^33^. Also in line with this study, we found that BA 7 appears to be relatively more co-activated than other associative areas.

The higher HC of primary regions compared to associative regions is possibly to be associated to the faster conduction callosal fibers connecting the primary cortices as well as to the slower fibers linking the associative areas^33,54,55^. Still, this could not be the only explanation. A direct relationship between structural and functional interhemispheric connectivity was not found in autistic patients with reduced callosal volume^160^ and individuals with callosal agenesis^161,162^, suggesting that other factors than callosal connectivity could shape functional HC. However, both autism and agenesis of the corpus callosum are congenital pathologies, in which neuronal plasticity could have reshaped interhemispheric connections^163–165^, allowing functional connectivity to be relatively preserved in absence of direct homotopic connections. When the CC is sectioned in adults, the impairment in functional interhemispheric connectivity are more evident^47,48,166^. Moreover, some degree of reduced inter-hemispheric connectivity has been found in both autism and callosal agenesis^52,53,160,167^. Therefore, it makes sense to relate functional HC mostly to callosal axons, even if also subcortical structures must be taken into account^168^. It remains unclear if local differences in functional HC strengths are explained by axonal speed, as proposed by Stark and colleagues^33^, or to other callosal regional features. For example, associative regions are characterized also by more distal FC than primary regions^169^. This could suggest that associative regions have a more diffuse connectivity, and therefore weaker homotopic FC could be explained by a less intense concentration of callosal homotopic connections in favor of more heterotopic callosal axons. However, to our best knowledge, no structural study has confirmed this hypothesis so far. Moreover, the higher number of connections exhibited by associative regions^170,171^ would allow them to have both many heterotopic connections and strong HC. On the contrary, the anatomical differences in axonal diameter and myelinization that differentiate the interhemispheric conduction speed of associative regions^54,56^ are sound structural findings that, in our opinion, can better account for the local differences in functional HC.

### Comparison between MHC and VMHC

Meta-analytical techniques like MACM or our MHC are based on the hypothesis that recurrent and statistically significant co-activation patterns are supposed to reveal connectivity pathways, expecting that the whole networks are functionally engaged by the appropriate tasks. This implies that these kinds of meta-analyses do not represent the rsFC, but the connectivity that is typical of active tasks, which is known to be partially divergent from that observed during rest^92,93,169,172–174^. To assess how HC changes from rest to task, we compared our results to those of the VMHC. The correlation value (r = 0.51) shows that meta-analytic homotopic co-activations reflect the intrinsic connectivity between hemispheres, but also points out that MHC is not a mirror image of VMHC.

Our VCC analysis produced a map that allows to discriminate between regions of convergence and divergence (Fig. 3). It is interesting to notice that such map presents several similarities with the map of global vs. local functional connectivity density by Tomasi and Volkow^175^ (see Fig. 6B of ref.^175^). Specifically, areas of greatest convergence in the VCC map are substantially superimposed to the regions more characterized by global, rather than local, functional connectivity density, which correspond to areas of high number of significant functional connections computed on the whole brain. Conversely, areas of high divergence in the VCC map are similar to regions in which local functional connectivity density, that is, regions of high number of significant functional connections computed considering only their closest neighbors, surpasses the global hubness. This could mean that those areas of strong global connectivity have a role in diffusion and integration of information that keeps their functional utility both in rest and task, while areas of higher local than global connectivity, which can be considered as peripheral hubs, are differently modulated if they are at rest or engaged in a task. Such modulation can consist in an increase of connectivity in rest compared to task or vice-versa, depending on their functional role. Tomasi and Volkow^175^ suggest that many areas of local hubness during rest could become more globally connected during task, as these areas display mostly executive functions along with attentional regions. To investigate if an area of divergence is characterized by stronger HC with co-activations or resting state signal correlation map, we calculated the voxel-wise difference between the two z-points standardized maps.

Certain areas reveal striking similarities (Fig. 6A), such as most part of the temporal lateral (BAs 20, 37, 38) and occipital cortices (BAs 17 and 18), or BA 31. Interestingly, other regions show different patterns of co-activations in the MHC or in the VMHC (Fig. 6B). Among the many areas showing the greatest difference in the MHC and in the VMHC are the subcortical nuclei, such as the anterior nucleus, the locus coeruleus (LC) and the medial geniculate body. Indeed, compared to the cortical areas, subcortical regions are known to show more difference in connectivity between the rest and task conditions^172^. More specifically, as predicted by Tomasi and Volkow^175^, they appear more involved in task than rest, that is, they tend to be more present in the MHC map than in the VMHC map. In fact, between the 25 areas preferentially revealed by the meta-analytic technique we found almost exclusively subcortical regions (Fig. 6A). Conversely, many cortical areas showed stronger homotopic rsFC than co-activation, such as BAs 1, 2, and 40 within the parietal regions; BAs 8, 45, 46 and 47 within the prefrontal regions; and the BAs 32 and 33 of the cingulate gyrus. Sepulcre and colleagues^169^ provided evidence that the execution of a task drives the activated regions to exhibit a more local, rather than distal, connectivity. This phenomenon could account for the dominance of medial regions in the MHC map and of lateral regions in VMHC map (Figs 4 and 7). Specifically, for regions close to the midline activations induce a stronger HC because they are close to their contralateral homologue, while lateral regions tend to present a relatively weaker HC because their homotopic areas are distant. Subcortical regions are placed in a medial position, so they can reveal stronger HC if activated by tasks. Functionality of LC has been directly associated with cognitive functions^176–178^ typically involved in experimental tasks, like modulation of salience^179^, attentional shifting^180–182^, attentional bias^183^, task performance optimization^184^, and memory^185,186^. Moreover, the noradrenaline released by LC is involved in working memory^187–190^ and in increasing the signal/noise ratio in primary sensory cortices^191–197^. Other strongly co-activated areas are the following: medial and the lateral geniculate bodies, which have auditory^198–202^ and visual^203–205^ functions, respectively; the substantia nigra, which is well known for its role in motor control^206–210^ but has been also thought to be implicated in cognitive activity^211–213^; and several regions of the cerebellum, which, too, are supposed to be involved both in motor and in cognitive functions^214–220^, such as executive control, working memory^221–225^, and attention^226,227^. The cognitive role of these medial subcortical regions may suggest that, during the performances of tasks, they should be functionally active, so as to have more local connectivity and, therefore, more HC.

It should be noted, however, that other subcortical regions appear to be more active in the VMHC than in the MHC map. A possible explanation is that these areas do not seem particularly involved in active tasks. For example, the anterior thalamic nucleus has been associated with episodic memory and is strongly connected with ACC, PCC/Rsp, inferior parietal lobule and especially the hippocampus^228,229^; these areas are parts of the default mode network (DMN), which is more active when the brain is at rest than when is engaged in a task^230,231^. In turn, the hypothalamus is involved in the control of homeostasis^232–234^, which cannot be operationalized by experimental task, and the caudate nucleus has been related to motor functions, as well as to behavior planning and control^235,236^, which may be difficult to investigate in the MRI scan, as motor tasks are generally simple manual actions.

Contrarily to what predicted by Tomasi and Volkow^175^, executive areas such as prefrontal lateral cortex and inferior parietal lobule reveal stronger values on the VMHC map, indicating that their HC remains stronger at rest compared to task, despite the obvious role of executive functions in task execution. Our study focuses only on HC, therefore it is possible that FC of executive regions is higher than that of DMN for what concerns intrahemispheric and heterotopic FC. Moreover, despite the obvious involvement of the executive network during task and DMN at rest, the fact that the latter network is placed medially, could possibly force its HC to be stronger than the executive areas during task execution as an effect of the task-induced augmentation of local connectivity. That is, DMN medial regions could be relatively more homotopically connected than executive lateral regions just as an effect of their greater proximity. In other words, it is likely that executive regions enhance their connectivity during task, but HC is a peculiar form of connectivity, that could be modulated by rest and task in a different way that global rsFC. As some involvement of DMN in task executions has already been postulated^237^, it could be possible that its role during task could be possibly mediated by HC, while the activity of executive regions could be relatively more sustained by inter-hemispheric or heterotopic connections, as if task engagement might require more inter-hemispherical collaboration for DMN than for executive regions.

Unfortunately, the effect of physical proximity cannot account for the greater involvement of the STG in MHC compared to VMHC as displayed by the difference map (Fig. 4). A behavioral analysis^119^ suggests that, with regard to the BrainMap database, the difference may be related to auditory perception as well as to linguistic functions (Fig. 8); it is not clear therefore whether or not the major involvement of this area in the MHC map is due to its activity in perceptual or higher cognitive functions. Also, it is possible that this result is spurious, as it can be caused by differences between the two databases.

### Limitations and future directions

Our MHC analysis is based on an anatomical atlas, so, differently form the VMHC, is not a voxel-wise technique. We chose this method in order to achieve a good statistical power as well as to reduce the noise in our data, as in voxel-wise meta-analyses some voxels can have an insufficient number of samples. However, this can diminish the spatial resolution of our map, thus potentially losing interesting details of local variability of HC.

Moreover, the symmetrization of the anatomical atlas can overcome potential biases induced by cerebral structural asymmetries, but, since these asymmetries could have functional meaning, they may have led to some extent to an incorrect identification of the patterns of intrinsic HC. It should be noted, however, that also the VMHC algorithm makes the symmetrization of the subjects’ anatomy^57^; our choice therefore do not differ much from the algorithms commonly used to compute HC. Moreover, it should also be considered that the afore-mentioned limitations can have only the effect of lowering the correlation value between MHC and VMHC, thus raising the possibility of producing false negatives rather than false positives. This should be reassuring, as it means that our technique is able to replicate the results of the VMHC.

Another possible confounding factor introduced by the utilization of an anatomical atlas emerges from the different dimension of the areas. The use of homogeneous parcellations has demonstrated that the results can be influenced by the different atlases and by the dimension of the parcels. However, the higher correlation between the VMHC and the MHC maps computed with an anatomical atlas compared to those made with the automatic parcellation, suggest that co-activations display a better convergence with FC if they are calculated using an atlas having an anatomical meaning rather than being constructed following the methodological assumption of homogeneous volume of the parcels.

Although age and sex have an effect on HC^57,86^, it must be observed that it was not possible to extract those variables from the meta-analytical data. In order to minimize the effect of sex, however, we randomly excluded a number of female subjects from the resting state dataset in order to get a 1:1 ratio. On the other hand, the effect of age could not be controlled in any way, since the resting state database was composed by young adults, while the data downloaded from BrainMap were likely to be obtained from subjects belonging to every age cohorts. The impossibility to take into account those variables constitute a limitation of the comparison between the meta-analytical and resting state HC techniques.

Another possible confounding aspect, which is however shared by all meta-analytic models, is that FC patterns are obtained from activation reports. Although meta-analytic co-activations mirror FC^89–91^, the two methods work on different data, and thus we cannot exclude the possibility that this difference would not bias to some extent the results. For example, perceptive cortices associated with a specific sensory modality in both hemispheres are likely to be activated at the same moment when an appropriate stimulus is presented. When we perceive, say, a sound, both our auditory cortices are activated independently of their reciprocal connectivity, which, given the scarcity of callosal projections between the temporal lobes, appears to be relatively moderate^46^. The experiments stored in the BrainMap database reporting foci in the auditory areas probably performed binaural auditory stimulations, which stimulated simultaneously both the left and right cortices, thus producing co-activation. Moreover, the acoustic noise produced during the scan by the MRI machine could increase the BOLD signal in bilateral auditory cortices not only in auditory tasks but in every scanning session, so as to inflate in the database the number of experiments that include bilateral auditory cortical foci. This phenomenon can somehow account for the greater involvement of the STG in the MHC map compared to the VMHC map. It should be observed, however, that having common inputs, as well as common outputs, drives two regions to be functionally connected^238^; thus, primary sensory and motor bilateral areas can present HC even in absence of a direct structural connection, since they both receive sensorial inputs from the thalamus and send motor command through the corticospinal tract. Therefore, it is unlikely that co-activations are present with a total lack of FC.

Apart from this specific and hypothetical issue on perceptive areas, it should be observed that meta-analytical models of FC express effects on parameters of connectivity that are not reproduced in studies that directly compare rest and task connectivity, both measured as BOLD time series correlations. For example, although Di *et al*.^239^ observed augmented efficiency and diminished small-worldness in a meta-analytical connectome compared to rsFC, Goparaju *et al*.^173^ found these metrics unchanged between rest and task connectivity. This could mean that the meta-analytical modeling of the FC could induce different biases to the research in task-related connectomics. Further investigations are therefore needed to clarify whether and in what extent the difference between meta-analytic models and resting state studies merely reflects the nature of co-activation analyses or true changes from rsFC evoked by task. Still, given the similarity between intrinsic connectivity and meta-analytic connectivity models, it seems likely that the supposed afore-mentioned biases would not strongly affect our results.

Finally, an open issue concerns the specific patterns of differences revealed by our research, and whether or not they are real or artifactual. For example, a close examination of the ACC reveals that BAs 32 and 33 are more involved in the MHC map than in the VMHC map, whereas BA 24 seems to be evenly present in the two maps, thus leading to think that functional differences between these areas depend on how specifically cytoarchitectonic partitions of the ACC are engaged in the performance of tasks. Future studies are needed to clarify this interesting issue and other questions about divergences between rsFC and meta-analytic co-activations, for instance with regard to pathological populations, or to investigate how gender, age or other variables can modulate HC during task performances. Another potential application of the MHC is to compare its results to those of structural covariance techniques, as some studies suggest that covariance in voxel-based morphometry^240^ or cortical thickness measured with surface-based morphometry^241,242^ are characterized by spatial patterns correlated with rsFC and meta-analytic co-activations^94^. With the help of MHC, it could also be interesting to test the changes of HC across a wide range of tasks.

## Conclusion

In this study we analyzed the entire BrainMap functional database of healthy subjects, thus modeling a theoretical task-general condition. We observed patterns of HC varying across brain regions (characterized by high connectivity in primary areas and a negative effect of Euclidean distance) that are generally consistent with those reported by Stark and colleagues^33^. The MHC analysis can therefore be a valuable tool to investigate HC in a meta-analytical fashion. Results obtained with this technique can support the findings of VMHC as well as of other meta-analytical models commonly used in connectivity studies. Meta-analytic connectivity models are not only useful to replicate rsFC, but also to investigate how connectivity changes from rest to task. Given the importance of interhemispheric interactions for brain functional processes, MHC may be useful to focus on task-induced transition of HC. We observed a series of differences between our MHC and the VMHC results that we interpreted as the effects of improved local connectivity during task and of regional variation in global hubness, with brain hubs more likely to show greater HC at rest. In particular, we have found that the regions which are proximal to the midline increase their HC during task, and that hubs are more connected in rest than in task, while primary regions maintain their typically high connectivity.

In conclusion, the MHC could prove itself useful as any other meta-analytic technique, like the MACM and the ICA executed on co-activation data, but focusing on the fascinating and important domain of HC. Therefore, it could be successfully used in connectivity studies with a broad range of applications.

## Supplementary information

The homotopic connectivity of the functional brain - Supplementary Materials
