# Supplementary material for: The homotopic connectivity of the functional brain: a meta-analytic approach
Source: Sci Rep. 2019 Mar 4;9:3346. doi: 10.1038/s41598-019-40188-3 (PMC6399443; doi:10.1038/s41598-019-40188-3)
Supplement: Supplementary file 1 — The homotopic connectivity of the functional brain - Supplementary Materials [file 41598_2019_40188_MOESM1_ESM.docx]

The homotopic connectivity of the functional brain:

a meta-analytic approach

Lorenzo Mancuso^2^, Tommaso Costa^1,2^, Andrea Nani^1,2^, Jordi Manuello^1,2^, Donato Liloia^1,2^,

Gabriele Gelmini^2^, Melissa Panero^2^, Sergio Duca^1^, Franco Cauda^1,2^

^1^ GCS-fMRI, Koelliker Hospital and Department of Psychology, University of Turin, Turin, Italy

^2^ Focus Lab, Department of Psychology, University of Turin, Turin, Italy

Supplementary Figures

**Figure S1 [PRISMA flow chart].** Overview of the selection strategy.

Algorithm:

*[Experiments Context IS Normal Mapping]*

*AND*

*[Experiments Activation IS Activations Only]*

*AND*

*[Subjects Diagnosis IS Normals]*

**2370 Functional Records** identified through **BrainMap**

Identification

**2370** Records after duplicates removed

Screening

**2370** Records screened

Studies were included if:

a) they were original studies published in a peer-reviewed English language journal;

b) they used any activation paradigm for functional imaging;

c) they adopted a whole-brain analysis;

d) they included only normal subjects;

e) the locations of activation were reported in Talairach/Tournoux (TAL) or in Montreal Neurological Institute (MNI) stereotaxic space.

**2370** Full-text assessed for eligibility

Eligibility

**2370 Functional studies** included in

quantitative synthesis

Included

**Figure S2.** Cortical surface mapping of the meta-analytic homotopic connectivity implemented with two alternative thresholds of activation. Values of correlation with the 20% threshold MHC are provided for each map.

**
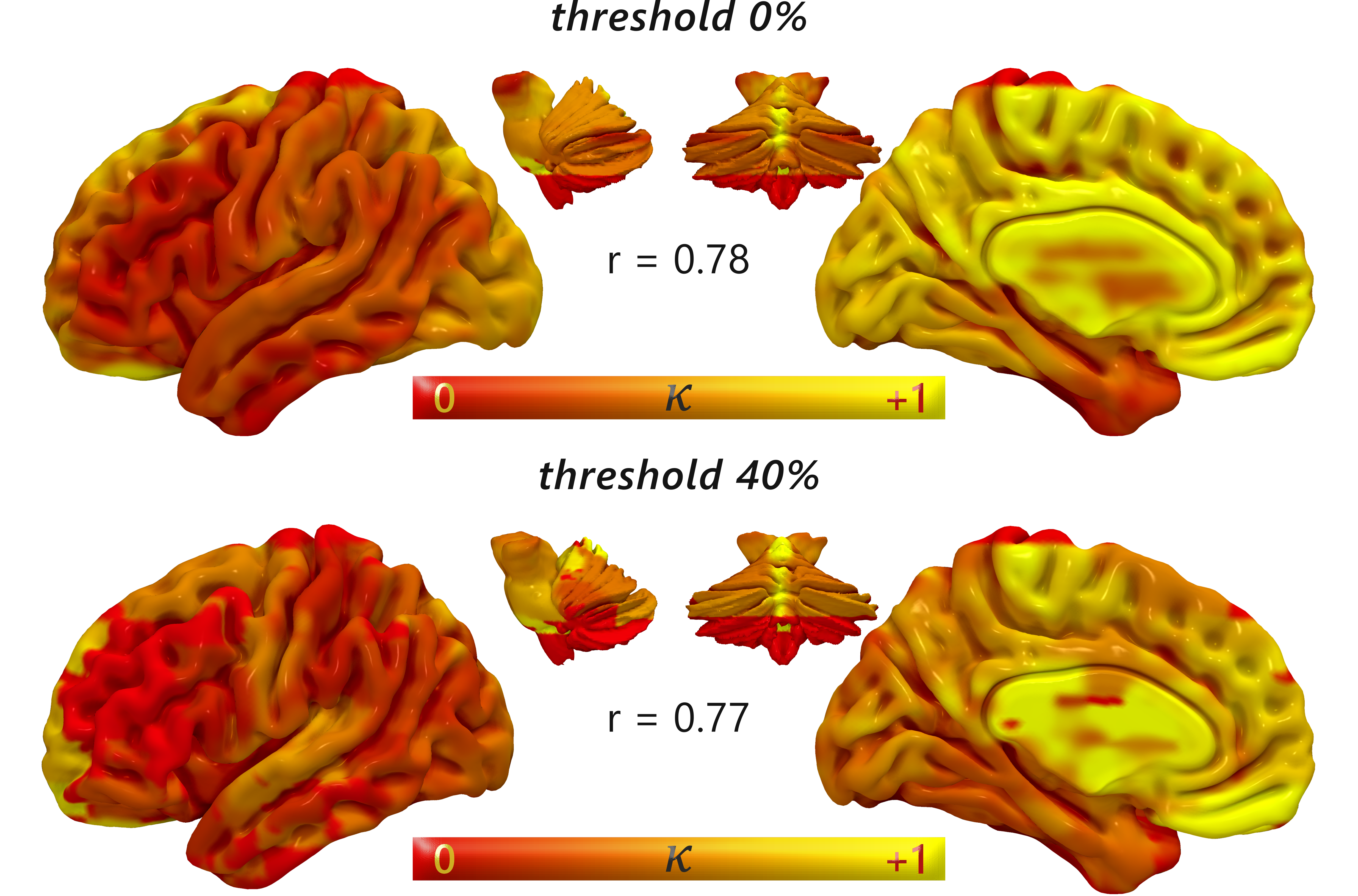
**

**Figure S3.** Cortical surface mapping of the voxel-mirrored homotopic connectivity. Colors from green to blue indicate the strength of homotopic connectivity.


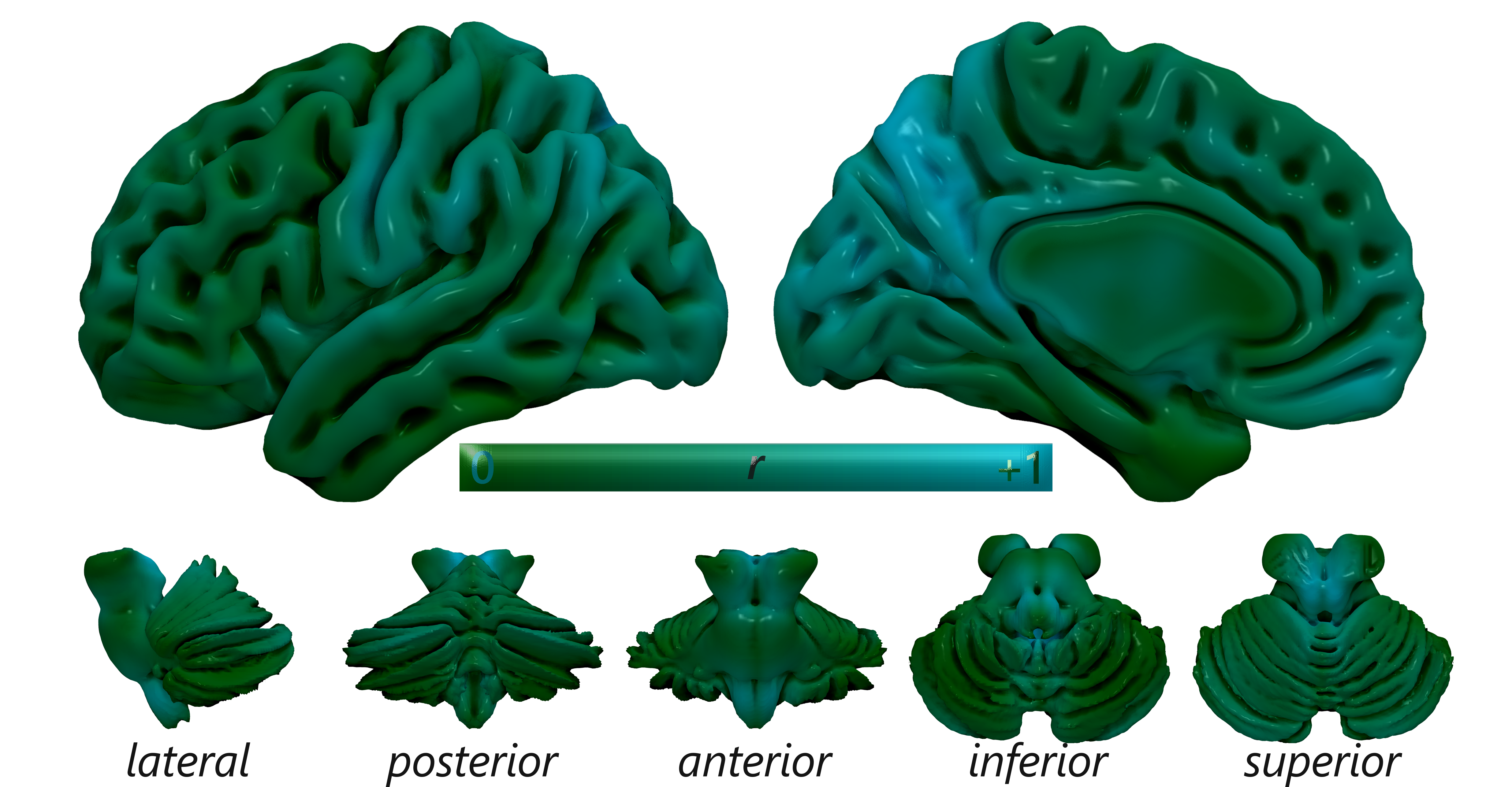


Supplementary Tables

**Table S1**. Types of paradigm classes with relative number of experiments and subjects for each paradigm class of the functional BrainMap database used in the analysis.

Experiments (N) = number of experiments; Experiments (%) = percentage of the total of the selected experiments; Subj (N) = number of subjects.

| ***Paradigm Class*** | ***Articles*** | ***Experiments*** | | ***Subj (N)*** |
| --- | --- | --- | --- | --- |
|  |  | ***(N)*** | ***(%)*** |  |
| Acupuncture | 7 | 33 | 0.3 | 193 |
| Affective pictures | 33 | 225 | 1.7 | 1326 |
| Affective words | 8 | 62 | 0.5 | 398 |
| Anti-Saccades | 8 | 31 | 0.2 | 202 |
| Chewing/swallowing | 15 | 90 | 0.7 | 397 |
| Classical conditioning | 17 | 90 | 0.7 | 377 |
| Competition/cooperation | 3 | 29 | 0.2 | 116 |
| Counting/calculation | 44 | 232 | 1.8 | 1116 |
| Cued Explicit Recognition/recall | 65 | 382 | 2.9 | 1791 |
| Deception | 12 | 52 | 0.4 | 340 |
| Delay discounting | 5 | 42 | 0.3 | 262 |
| Dealyed match to sample | 48 | 259 | 2.0 | 1397 |
| Divided auditory attention | 4 | 24 | 0.2 | 98 |
| Drawing | 4 | 39 | 0.3 | 77 |
| Driving | 3 | 15 | 0.1 | 69 |
| Emotion induction | 81 | 462 | 3.5 | 2790 |
| Emotional body language perception | 2 | 17 | 0.1 | 51 |
| Encoding | 48 | 236 | 1.8 | 1489 |
| Episodic recall | 22 | 117 | 0.9 | 554 |
| Estimation | 4 | 15 | 0.1 | 120 |
| Face monitoring/discrimination | 97 | 573 | 4.4 | 2755 |
| Figurative language | 6 | 33 | 0.3 | 160 |
| Film viewing | 42 | 266 | 2.0 | 1199 |
| Finger tapping/button press | 193 | 1177 | 9.0 | 7011 |
| Fixation | 9 | 30 | 0.2 | 185 |
| Flanker | 10 | 37 | 0.3 | 266 |
| Flashing checkerboard | 3 | 33 | 0.3 | 185 |
| Flexion/extension | 34 | 199 | 1.5 | 790 |
| Fluency induction | 1 | 3 | 0.0 | 12 |
| Free list word record | 4 | 11 | 0.1 | 69 |
| Gambling | 22 | 159 | 1.2 | 702 |
| Go/No go | 50 | 246 | 1.9 | 1841 |
| Grasping | 9 | 45 | 0.3 | 146 |
| Hand-Eye Coordination | 2 | 7 | 0.1 | 44 |
| Hunger/satiety | 6 | 26 | 0.2 | 134 |
| Hypercapnia /air hunger | 7 | 22 | 0.2 | 104 |
| Imagined movement | 20 | 94 | 0.7 | 522 |
| Imagined objects/scenes | 25 | 128 | 1.0 | 742 |
| Induced panic | 2 | 6 | 0.0 | 62 |
| Isometric force | 5 | 26 | 0.2 | 104 |
| Lexical decision | 6 | 26 | 0.2 | 165 |
| Magnitude comparison (distance) | 1 | 15 | 0.1 | 38 |
| Magnitude comparison (luminance) | 2 | 13 | 0.1 | 37 |
| Magnitude comparison (numerical) | 2 | 13 | 0.1 | 37 |
| Magnitude comparison (phisical size) | 3 | 20 | 0.2 | 97 |
| Magnitude comparison (symbolic) | 5 | 24 | 0.2 | 126 |
| Meditation | 11 | 85 | 0.6 | 433 |
| Mental rotation | 19 | 117 | 0.9 | 493 |
| Micturition | 6 | 25 | 0.2 | 128 |
| Motor learning | 1 | 7 | 0.1 | 15 |
| Multi tasking | 3 | 14 | 0.1 | 82 |
| Music comprehension | 19 | 155 | 1.2 | 510 |
| Music production | 17 | 77 | 0.6 | 407 |
| N-back | 64 | 291 | 2.2 | 2609 |
| Naming (covert) | 14 | 73 | 0.6 | 288 |
| Naming (overt) | 20 | 120 | 0.9 | 467 |
| Object manipulation/discrimination | 4 | 32 | 0.2 | 58 |
| Oddball discrimination | 12 | 48 | 0.4 | 376 |
| Olfactory monitoring/discrimination | 22 | 141 | 1.1 | 557 |
| Orthographic discrimination | 24 | 124 | 0.9 | 653 |
| Pain monitor/discrimination | 63 | 325 | 2.5 | 1439 |
| Paired associate recall | 27 | 138 | 1.0 | 699 |
| Passive listening | 48 | 234 | 1.8 | 1153 |
| Passive viewing | 79 | 450 | 3.4 | 2186 |
| Phonological discrimination | 38 | 209 | 1.6 | 857 |
| Pitch monitor/discrimination | 22 | 142 | 1.1 | 554 |
| Pointing | 8 | 34 | 0.3 | 113 |
| Pursuit rotor/manual tracking | 4 | 21 | 0.2 | 75 |
| Reading (covert) | 37 | 194 | 1.5 | 872 |
| Reading (overt) | 26 | 131 | 1.0 | 595 |
| Reasoning/problem solving | 36 | 206 | 1.6 | 1318 |
| Recitation/repetition (covert) | 10 | 31 | 0.2 | 212 |
| Recitation/repetition (overt) | 19 | 78 | 0.6 | 408 |
| Rest | 11 | 36 | 0.3 | 386 |
| Reward | 106 | 728 | 5.5 | 3432 |
| Saccades | 31 | 123 | 0.9 | 574 |
| Self-reflection | 2 | 24 | 0.2 | 87 |
| Semantic monitor/discrimination | 109 | 616 | 4.7 | 2960 |
| Sequence recall/learning | 14 | 65 | 0.5 | 334 |
| Sexual arousal/gratification | 17 | 122 | 0.9 | 609 |
| Sleep | 3 | 7 | 0.1 | 77 |
| Stroop - color | 30 | 110 | 0.8 | 1049 |
| Stroop - counting | 4 | 14 | 0.1 | 105 |
| Stroop - emotional | 8 | 23 | 0.2 | 328 |
| Stroop - other | 3 | 14 | 0.1 | 103 |
| Stroop - spatial | 2 | 10 | 0.1 | 36 |
| Syntactic discrimination | 8 | 23 | 0.2 | 212 |
| Tactile monitor/discrimination | 28 | 134 | 1.0 | 580 |
| Task switching | 22 | 117 | 0.9 | 644 |
| Taste | 18 | 113 | 0.9 | 623 |
| Theory of mind | 38 | 253 | 1.9 | 1197 |
| Thirst induction | 3 | 11 | 0.1 | 67 |
| Tone monitor/discrimination | 27 | 119 | 0.9 | 643 |
| Tower of London | 4 | 15 | 0.1 | 84 |
| Transcranical Magnetic Stimulation | 8 | 35 | 0.3 | 129 |
| Trauma recall | 2 | 7 | 0.1 | 68 |
| Vestibular Stimulation | 3 | 7 | 0.1 | 54 |
| Vibrotactile monitor/discrimination | 8 | 130 | 1.0 | 169 |
| Video games | 4 | 20 | 0.2 | 111 |
| Visual Motion | 2 | 11 | 0.1 | 55 |
| Visual object identification | 33 | 229 | 1.7 | 853 |
| Visual pursuit/tracker | 19 | 95 | 0.7 | 390 |
| Visuospatial attention | 70 | 353 | 2.7 | 1693 |
| Wisconsin card sorting test | 12 | 57 | 0.4 | 352 |
| Word generation (covert) | 35 | 155 | 1.2 | 779 |
| Word generation (overt) | 28 | 142 | 1.1 | 782 |
| Word imageability | 3 | 13 | 0.1 | 84 |
| Word stem completion (covert) | 2 | 6 | 0.0 | 64 |
| Word Stem completion (overt) | 4 | 14 | 0.1 | 103 |
| Writing | 3 | 11 | 0.1 | 83 |
| ***TOTAL*** | ***2370*** | ***13148*** | ***100%*** | ***68152*** |

**Table S2.** Comparison between the mean values of co-activation obtained for each brain region using different atlases

| **Cerebral regions** | **Mean Patel’s κ of TD MHC** | **Mean Patel’s κ of k=997 MHC** | **Mean Patel’s κ of k=250 MHC** |
| --- | --- | --- | --- |
| Amygdala | 0.421945 | 0.386372 | 0.457857 |
| Anterior Nucleus (of thalamus) | 0.383005 | 0.262508 | 0.394543 |
| BA 1 (S1) | 0.185581 | 0.240488 | 0.243172 |
| BA 2 (S1) | 0.250361 | 0.219607 | 0.19973 |
| BA 3 (S1) | 0.274252 | 0.215666 | 0.231563 |
| BA 4 (M1) | 0.303344 | 0.191099 | 0.212297 |
| BA 5 (somatosensory association cortex) | 0.37231 | 0.20022 | 0.190503 |
| BA 6 (premotor and supplementary motor cortex) | 0.211299 | 0.276117 | 0.267671 |
| BA 7 (precuneus) | 0.373143 | 0.152818 | 0.149203 |
| BA 8 (dorsolateral prefrontal cortex) | 0.181737 | 0.231354 | 0.28821 |
| BA 9 (dorsolateral prefrontal cortex) | 0.172451 | 0.156401 | 0.153193 |
| BA 10 (anterior prefrontal cortex) | 0.203801 | 0.138897 | 0.181426 |
| BA 11 (orbitofrontal cortex) | 0.126643 | 0.103134 | 0.15767 |
| ant BA 13 (anterior insula) | 0.26194 | 0.189321 | 0.209396 |
| post BA 13 (posterior insula) | 0.278194 | 0.245099 | 0.312552 |
| BA 17 (V1) | 0.437367 | 0.255032 | 0.285657 |
| BA 18 (V2) | 0.398311 | 0.196103 | 0.209484 |
| BA 19 (V3, V4, V5) | 0.310256 | 0.142368 | 0.155508 |
| BA 20 (inferior temporal gyrus) | 0.209217 | 0.09702 | 0.075704 |
| BA 21 (middle temporal gyrus) | 0.184597 | 0.035834 | 0.033972 |
| BA 22 (superior temporal gyrus) | 0.35691 | 0.087318 | 0.128591 |
| BA 23 (ventral posterior cingulate cortex | 0.419182 | 0.231797 | 0.267807 |
| BA 24 (ventral anterior cingulate cortex) | 0.390837 | 0.369858 | 0.444303 |
| BA 25 (subgenual cortex) | 0.387982 | 0.250929 | 0.285676 |
| BA 27 (piriform cortex) | 0.286465 | 0.218106 | 0.230159 |
| BA 28 (ventral entorhinal cortex) | 0.379729 | 0.27653 | 0.311358 |
| BA 29 (retrosplenial cortex) | 0.469588 | 0.080612 | 0.10285 |
| BA 30 (retrosplenial cortex) | 0.435394 | 0.194315 | 0.218101 |
| BA 31 (dorsal posterior cingulate cortex) | 0.446719 | 0.313068 | 0.348653 |
| BA 32 (dorsal anterior cingulate cortex) | 0.45908 | 0.326495 | 0.40067 |
| BA 33 (pregenual cortex) | 0.441305 | 0.151074 | 0.260665 |
| BA 34 (dorsal entorhinal cortex) | 0.417744 | 0.383554 | 0.407055 |
| BA 35 (perirhinal cortex) | 0.383396 | 0.23009 | 0.263072 |
| BA 36 (ectorhinal cortex) | 0.351094 | 0.251667 | 0.263517 |
| BA 37 (fusiform gyrus) | 0.262479 | 0.069478 | 0.106594 |
| BA 38 (temporal pole) | 0.208062 | 0.081085 | 0.077409 |
| BA 39 (angular gyrus) | 0.237625 | 0.135684 | 0.126935 |
| BA 40 (supramarginal gyrus) | 0.206721 | 0.125418 | 0.136674 |
| BA 41 (Heschl’s gyrus) | 0.48804 | 0.17374 | 0.248533 |
| BA 42 (Heschl’s gyrus) | 0.323032 | 0.125166 | 0.144477 |
| BA 43 (primary gustatory cortex) | 0.331811 | 0.13861 | 0.150584 |
| BA 44 (pars opercularis) | 0.154006 | 0.079357 | 0.09618 |
| BA 45 (pars triangularis) | 0.072601 | 0.06317 | 0.062951 |
| BA 46 (middle frontal gyrus) | 0.063626 | 0.091818 | 0.101577 |
| BA 47 (pars orbitalis) | 0.07704 | 0.171892 | 0.159558 |
| Caudate Body | 0.352557 | 0.126888 | 0.177275 |
| Caudate Head | 0.445158 | 0.181272 | 0.207077 |
| Caudate Tail | 0.119509 | 0.267161 | 0.236519 |
| Cerebellar Lingual | 0.440578 | 0.527997 | 0.459716 |
| Cerebellar Tonsil | 0.032251 | 0.061923 | 0.057325 |
| Culmen (of cerebellum) | 0.389972 | 0.047702 | 0.052413 |
| Culmen of Vermis (of cerebellum) | 0.31011 | 0.441535 | 0.466411 |
| Declive (of cerebellum) | 0.333633 | 0.280003 | 0.333561 |
| Declive of Vermis (of cerebellum) | 0.26315 | 0.533369 | 0.604062 |
| Dentate (of cerebellum) | 0.438839 | 0.274866 | 0.299615 |
| Fastgium (of cerebellum) | 0.447759 | 0.303139 | 0.316671 |
| Hippocampus | 0.273088 | 0.317316 | 0.312237 |
| Hypotalamus | 0.203815 | 0.319243 | 0.396254 |
| Inferior Semi-Lunar Lobule (of cerebellum) | 0.178091 | 0.223959 | 0.237283 |
| Lateral Geniculate Body (of thalamus) | 0.254908 | 0.443251 | 0.431329 |
| Lateral Globus Pallidus | 0.416523 | 0.329888 | 0.340861 |
| Lateral Posterior Nucleus (of thalamus) | 0.302745 | 0.177698 | 0.178336 |
| Lateral Dorsal Nucleus (of thalamus) | 0.415429 | 0.356106 | 0.257805 |
| Locus Coeruleus | 0.53714 | 0.358378 | 0.551883 |
| Mammillary Body | 0.433164 | 0.303985 | 0.353417 |
| Medial Dorsal Nucleus (of thalamus) | 0.498281 | 0.218481 | 0.274267 |
| Medial Globus Pallidus | 0.37996 | 0.277723 | 0.390815 |
| Medial Geniculate Body (of thalamus) | 0.46404 | 0.101329 | 0.124643 |
| Medulla Oblongata | 0.171175 | 0.226155 | 0.25429 |
| Midline Nucleus (of thalamus) | 0.447163 | 0.368332 | 0.388025 |
| Nodule (of cerebellum) | 0.520642 | 0.129619 | 0.134909 |
| Nucleus Accumbens | 0.4239 | 0.49655 | 0.42654 |
| Periaqueductal gray | 0.315749 | 0.658849 | 0.663481 |
| Pons | 0.331233 | 0.407595 | 0.484199 |
| Pulvinar | 0.291395 | 0.168969 | 0.179385 |
| Putamen | 0.332826 | 0.371165 | 0.364077 |
| Pyramis (of cerebellum) | 0.256187 | 0.198333 | 0.214355 |
| Pyramis of Vermis (of cerebellum) | 0.291821 | 0.086781 | 0.149381 |
| Red Nucleus | 0.474307 | 0.544548 | 0.555755 |
| Substantia Nigra | 0.498737 | 0.368395 | 0.440723 |
| Subthalamic Nucleus | 0.404303 | 0.380856 | 0.46979 |
| Tuber (of cerebellum) | 0.147629 | 0.015583 | 0.016235 |
| Tuber of Vermis (of cerebellum) | 0.249696 | 0.179975 | 0.282427 |
| Uvula (of cerebellum) | 0.285294 | 0.244681 | 0.273366 |
| Uvula of Vermis (of cerebellum) | 0.214783 | 0.408058 | 0.493733 |
| Ventral Lateral Nucleus (of thalamus) | 0.38251 | 0.180321 | 0.31881 |
| Ventral Anterior Nucleus (of thalamus) | 0.289996 | 0.335546 | 0.356313 |
| Ventral Posterior Lateral Nucleus (of thalamus) | 0.336847 | 0.335551 | 0.321717 |
| Ventral Posterior Medial Nucleus (of thalamus) | 0.411369 | 0.263815 | 0.275483 |

**Table S3.** Mean co-activation, correlation, LOVOC values and difference between mean co-activation and mean correlation for each brain region.

| **Cerebral regions** | **MHC Mean Patel’s κ** | **VMHC Mean Pearson’s r** | **Mean LOVOC values** | **Mean values of the difference map** |
| --- | --- | --- | --- | --- |
| Amygdala | 0.421945 | 0.34115 | -0.09569 | 0.095182 |
| Anterior Nucleus (of thalamus) | 0.383005 | 0.59281 | 0.11653 | -0.22044 |
| BA 1 (S1) | 0.185581 | 0.340623 | -0.13249 | -0.1644 |
| BA 2 (S1) | 0.250361 | 0.373908 | -0.09718 | -0.12887 |
| BA 3 (S1) | 0.274252 | 0.354055 | -0.0598 | -0.08031 |
| BA 4 (M1) | 0.303344 | 0.383099 | -0.03703 | -0.08254 |
| BA 5 (somatosensory association cortex) | 0.37231 | 0.364312 | -0.15768 | 0.0108 |
| BA 6 (premotor and supplementary motor cortex) | 0.211299 | 0.282318 | -0.19548 | -0.07433 |
| BA 7 (precuneus) | 0.373143 | 0.48251 | 0.076194 | -0.12035 |
| BA 8 (dorsolateral prefrontal cortex) | 0.181737 | 0.315374 | -0.22414 | -0.14567 |
| BA 9 (dorsolateral prefrontal cortex) | 0.172451 | 0.290707 | -0.21867 | -0.12434 |
| BA 10 (anterior prefrontal cortex) | 0.203801 | 0.319298 | -0.20831 | -0.12342 |
| BA 11 (orbitofrontal cortex) | 0.126643 | 0.160101 | -0.11618 | -0.0322 |
| ant BA 13 (anterior insula) | 0.26194 | 0.303837 | -0.07459 | -0.03907 |
| post BA 13 (posterior insula) | 0.278194 | 0.267177 | -0.07325 | 0.019304 |
| BA 17 (V1) | 0.437367 | 0.464633 | 0.356441 | -0.02954 |
| BA 18 (V2) | 0.398311 | 0.393511 | 0.246743 | 0.004374 |
| BA 19 (V3, V4, V5) | 0.310256 | 0.367084 | 0.094138 | -0.0594 |
| BA 20 (inferior temporal gyrus) | 0.209217 | 0.189308 | -0.24665 | 0.028293 |
| BA 21 (middle temporal gyrus) | 0.184597 | 0.264573 | -0.15894 | -0.07961 |
| BA 22 (superior temporal gyrus) | 0.35691 | 0.294973 | -0.02968 | 0.067111 |
| BA 23 (ventral posterior cingulate cortex | 0.419182 | 0.519932 | 0.102111 | -0.11589 |
| BA 24 (ventral anterior cingulate cortex) | 0.390837 | 0.324583 | -0.1028 | 0.06884 |
| BA 25 (subgenual cortex) | 0.387982 | 0.326987 | -0.15597 | 0.06186 |
| BA 27 (piriform cortex) | 0.286465 | 0.311227 | -0.12319 | -0.02802 |
| BA 28 (ventral entorhinal cortex) | 0.379729 | 0.260391 | -0.30273 | 0.132512 |
| BA 29 (retrosplenial cortex) | 0.469588 | 0.513322 | 0.259786 | -0.05289 |
| BA 30 (retrosplenial cortex) | 0.435394 | 0.489804 | 0.241345 | -0.06247 |
| BA 31 (dorsal posterior cingulate cortex) | 0.446719 | 0.42966 | 0.006632 | 0.012964 |
| BA 32 (dorsal anterior cingulate cortex) | 0.45908 | 0.301097 | -0.14386 | 0.16655 |
| BA 33 (pregenual cortex) | 0.441305 | 0.308869 | -0.15217 | 0.144792 |
| BA 34 (dorsal entorhinal cortex) | 0.417744 | 0.370419 | 0.003852 | 0.048679 |
| BA 35 (perirhinal cortex) | 0.383396 | 0.264409 | -0.26454 | 0.132339 |
| BA 36 (ectorhinal cortex) | 0.351094 | 0.196479 | -0.39198 | 0.170459 |
| BA 37 (fusiform gyrus) | 0.262479 | 0.285989 | -0.12371 | -0.01937 |
| BA 38 (temporal pole) | 0.208062 | 0.215397 | -0.22379 | -0.00115 |
| BA 39 (angular gyrus) | 0.237625 | 0.340104 | -0.05109 | -0.10888 |
| BA 40 (supramarginal gyrus) | 0.206721 | 0.397127 | -0.15216 | -0.20027 |
| BA 41 (Heschl’s gyrus) | 0.48804 | 0.377986 | 0.0878 | 0.121039 |
| BA 42 (Heschl’s gyrus) | 0.323032 | 0.376569 | 0.114294 | -0.05461 |
| BA 43 (primary gustatory cortex) | 0.331811 | 0.349741 | 0.043352 | -0.01617 |
| BA 44 (pars opercularis) | 0.154006 | 0.260872 | -0.1983 | -0.10919 |
| BA 45 (pars triangularis) | 0.072601 | 0.200283 | -0.14191 | -0.12999 |
| BA 46 (middle frontal gyrus) | 0.063626 | 0.194271 | -0.12522 | -0.13511 |
| BA 47 (pars orbitalis) | 0.07704 | 0.233013 | -0.17731 | -0.15975 |
| Caudate Body | 0.352557 | 0.411467 | 0.057123 | -0.06416 |
| Caudate Head | 0.445158 | 0.436497 | 0.140894 | 0.005863 |
| Caudate Tail | 0.119509 | 0.254686 | -0.16023 | -0.13395 |
| Cerebellar Lingual | 0.440578 | 0.333873 | -0.07773 | 0.114615 |
| Cerebellar Tonsil | 0.032251 | 0.122945 | -0.03193 | -0.08271 |
| Culmen (of cerebellum) | 0.389972 | 0.27944 | -0.21789 | 0.125206 |
| Culmen of Vermis (of cerebellum) | 0.31011 | 0.297614 | -0.14206 | 0.012846 |
| Declive (of cerebellum) | 0.333633 | 0.310426 | -0.02651 | 0.029845 |
| Declive of Vermis (of cerebellum) | 0.26315 | 0.260374 | -0.20017 | 0.004049 |
| Dentate (of cerebellum) | 0.438839 | 0.227348 | -0.46317 | 0.236269 |
| Fastgium (of cerebellum) | 0.447759 | 0.305945 | -0.26427 | 0.161492 |
| Hippocampus | 0.273088 | 0.209274 | -0.20324 | 0.072573 |
| Hypotalamus | 0.203815 | 0.353494 | -0.32044 | -0.17066 |
| Inferior Semi-Lunar Lobule (of cerebellum) | 0.178091 | 0.168714 | -0.18039 | 0.01785 |
| Lateral Geniculate Body (of thalamus) | 0.415429 | 0.457876 | 0.135423 | -0.05616 |
| Lateral Globus Pallidus | 0.254908 | 0.150417 | -0.27566 | 0.106323 |
| Lateral Posterior Nucleus (of thalamus) | 0.416523 | 0.278761 | -0.1888 | 0.147188 |
| Lateral Dorsal Nucleus (of thalamus) | 0.302745 | 0.346522 | -0.08246 | -0.04489 |
| Locus Coeruleus | 0.53714 | 0.297436 | 0.44915 | 0.263916 |
| Mammillary Body | 0.433164 | 0.348817 | -0.02735 | 0.086086 |
| Medial Dorsal Nucleus (of thalamus) | 0.498281 | 0.49147 | 0.333902 | 0.008464 |
| Medial Globus Pallidus | 0.46404 | 0.209509 | -0.33536 | 0.282387 |
| Medial Geniculate Body (of thalamus) | 0.37996 | 0.208087 | -0.39709 | 0.180218 |
| Medulla Oblongata | 0.171175 | 0.318698 | -0.46486 | -0.14941 |
| Midline Nucleus (of thalamus) | 0.447163 | 0.486443 | 0.269711 | -0.03913 |
| Nodule (of cerebellum) | 0.520642 | 0.296818 | -0.29018 | 0.244744 |
| Nucleus Accumbens | 0.4239 | 0.519112 | 0.18551 | -0.12768 |
| Periaqueductal gray | 0.315749 | 0.351428 | -0.24923 | -0.03503 |
| Pons | 0.331233 | 0.260347 | -0.46283 | 0.080065 |
| Pulvinar | 0.291395 | 0.331637 | -0.08877 | -0.0423 |
| Putamen | 0.332826 | 0.357644 | 0.007388 | -0.02621 |
| Pyramis (of cerebellum) | 0.256187 | 0.22197 | -0.08371 | 0.04162 |
| Pyramis of Vermis (of cerebellum) | 0.291821 | 0.221492 | -0.23208 | 0.074491 |
| Red Nucleus | 0.474307 | 0.382809 | -0.06191 | 0.096028 |
| Substantia Nigra | 0.498737 | 0.262162 | -0.35477 | 0.257773 |
| Subthalamic Nucleus | 0.404303 | 0.239898 | -0.2869 | 0.178111 |
| Tuber (of cerebellum) | 0.147629 | 0.142628 | -0.04197 | 0.010395 |
| Tuber of Vermis (of cerebellum) | 0.249696 | 0.218218 | -0.19312 | 0.041322 |
| Uvula (of cerebellum) | 0.285294 | 0.248923 | -0.10448 | 0.046714 |
| Uvula of Vermis (of cerebellum) | 0.214783 | 0.251056 | -0.23615 | -0.04477 |
| Ventral Lateral Nucleus (of thalamus) | 0.289996 | 0.427329 | -0.0943 | -0.14322 |
| Ventral Anterior Nucleus (of thalamus) | 0.38251 | 0.337185 | -0.02057 | 0.048175 |
| Ventral Posterior Lateral Nucleus (of thalamus) | 0.336847 | 0.22889 | -0.1146 | 0.120572 |
| Ventral Posterior Medial Nucleus (of thalamus) | 0.411369 | 0.296618 | -0.04022 | 0.128923 |
